# Evaluation of ionic liquids based imidazolium salts as an environmentally friendly corrosion inhibitors for carbon steel in HCl solutions

**DOI:** 10.1038/s41598-024-52174-5

**Published:** 2024-01-22

**Authors:** Raghda A. El-Nagar, N. A. Khalil, Y. Atef, Maher I. Nessim, Alaa Ghanem

**Affiliations:** 1 Petroleum Testing Lab, Analysis and Evaluation Department, Egyptian Petroleum Research Institute, Nasr City, Cairo, 11727 Egypt; 2 Research Laboratory, General Organization for Export and Import Control (G.O.E.I.C), Cairo, Egypt; 3 PVT Lab, Production Department, Egyptian Petroleum Research Institute, Nasr City, Cairo, 11727 Egypt; 4 PVT Services Center, Egyptian Petroleum Research Institute, Nasr City, Cairo, 11727 Egypt

**Keywords:** Corrosion, Ionic liquids

## Abstract

The features of this work on corrosion inhibition have been investigated based on the ecological awareness and according to the strict environmental legislations. This was done by studying how different imidazolium derivatives ionic liquids containing different alkyl chains R_8_, R_10_ and R_12_ affected the corrosion reaction of carbon steel specimen immersed in 1 M hydrochloric acid at various temperatures. Weight loss, potentiodynamic polarization and electrochemical impedance spectroscopy were utilized to examine the corrosion inhibition behavior on carbon steel. In addition, FT-IR spectroscopy was used to analyze the coated film that has been formed on the metal surface. The prepared ionic liquids showed effective inhibition efficiency, where the corrosion rate after the using of 100 ppm of R_8_-IL, R_10_-IL and R_12_-IL was decreased from 5.95 (µg cm^−2^ min^−1^) to 0.66, 0.56, and 0.44 (µg cm^−2^ min^−1^), respectively at 20 °C. In the polarization curves, the corrosion current, I_corr_, decreases by ILs addition and suggest that ILs act as mixed type inhibitors. From EIS findings, the increase in R_ct_ and decrease in C_dl_ values proves the adherence of inhibitor molecules on carbon steel surface. The temperature effect was also studied on the film formed, where increasing the temperature from 20 to 50 °C, the corrosion rate increased and the inhibitors efficacy decreased. The increasing in the length of the attached alkyl chain, the efficacies of the prepared inhibitors increases. Various thermodynamic parameters such as the reaction activation free energy (ΔG^*^), the entropy of activation (ΔS^*^), and the enthalpy of activation (ΔH^*^), as well as the adsorption isotherm were investigated in order to interpret the mechanism and obtain the most accurate perception.

## Introduction

Carbon steel has superior mechanical and chemical qualities, therefore it is mostly used in several industrial applications, such as pipeline, construction, and implementations in petroleum industries^1^. On the other hand, its susceptibility and extreme sensitive to corrosion is one of the important problems of using carbon steel^2–4^, especially given how easily an acidic medium corrodes it. Moreover, the exploitation of harsh mineral acid conditions in the production of oil and gas to remove sand, scales, blocking sludge, and etching of carbon steel of corrosive scales, sludge, and dust, and etching of carbon steel alloys^5^. It is greatly impacted by these operations, particularly the use of hydrochloric acid, with enormous loss in resources and money^6^, leading to large economic issues in the production of oil and gas^7–9^. Some of these processes are undertaken by pushing certain concentrations of the hydrochloric acid solutions inside the oil reservoir in order to open certain channels and increase the formation porosity and permeability to enhance the oil flowability^10^. Besides of the highly destructive corrosion effect of hydrochloric acid medium, there are other problems in acids contacting such as the acid pickling procedures, which is a typical industrial operation that is used in the different activities in oilfields and the manufacturing of petrochemicals^11^. Therefore, the controlling of the corrosion process has much attention in different fields that are worked by carbon steel in both near neutral^12–14^ and acidic media^15–18^.

For the severe environmental restrictions, the utilization of green corrosion inhibitors is highly eligible. The production of safe and ecofriendly corrosion inhibitors is being pursued. Therefore, ionic liquids (ILs) have emerged as essential eco-friendly and cost-effective inhibitors that have garnered significant interest from both the academic researchers and the engineers^19–21^. The leverage of ionic liquids in many applications including corrosion inhibitors is mostly related to their physical–chemical properties such as high solubility, non-toxicity, low volatility, non-flammable, synthesis without solvents with a high yield, no-electronic conductivity, high thermal stability^22–24^. Therefore, it was worthy to discuss additional significant properties that have been linked to corrosion inhibitors that are environmentally benign and safe in different media. In addition, the chemical structure such as the alkyl chain length that may have a positive performance on the inhibition of corrosion process^21,25^; also, the formulated with π-electrons of double bonds as in benzene ring, and the lone pair of electrons on heteroatoms such as N, are well known as acid inhibitors. The presence of centers with high electron densities that permit the adsorption of the inhibitor molecules over the surface of metal and prevent them from any corrosive surroundings^21,26^. The chosen types of ILs in this study contains various active sites to form the most promising subclass depending on counter ions and nitrogen atoms which control their properties such as melting point, conductivity, solubility and viscosity^27^. Imidazolium ILs as heterocyclic nuclei containing nitrogen in their structures are diverse class of ILs which were recently included and attracted the attention because of their ability to create cationic molten salts. Moreover, imdazolieum based ILs were recorded as ecofriendly sustainable compounds in different industrial applications regarding to many properties such as biodegradability, non-toxicity, cost effectiveness, safety and their high solubility in water^28,29^. Gobara et. al, studied the application of some ILs as green inhibitors for the corrosion of carbon steel in acidic medium^19^, and the obtained results represented excellent efficiency of poly IL based acrylates. Also ILs based on fatty acids was studied in produced oilfield water by Atef et al. to investigate the effect of the fatty acid chain length^21^. It was found that the prepared DEA-C_n_ ILs was efficient as green inhibitors for the corrosion of carbon steel; moreover, their efficacy increase by increasing the chain length of the fatty acids. In this study, we mainly aimed to study the electrochemical tests, weight loss measurements and metal surface studies to examine the effect of 3-(4-methylbenzyl)-1-octyl-1-H-imidazolium chloride, 3-(4-methylbenzyl)-1-decyl-1-H-imidazolium chloride and 3-(4-methylbenzyl)-1-dodecyl-1-H-imidazolium chloride as inhibitors for the corrosion of carbon steel in 1 M hydrochloric acid. The performance of the synthesized ILs was determined through studying the effect of temperature, concentrations and the difference in their chemical structure as the alkyl chain length.

## Experimental

### Materials

The experimental evaluation of the prepared ILs were carried out on carbon steel specimens that composed of Carbon = 0.12%, Manganese = 0.5%, Sulfur = 0.6%, Silicon = 0.17%, Phosphorus = 0.04%, and the remainder is Iron.

### Preparation of ILs

**Figure 1:**
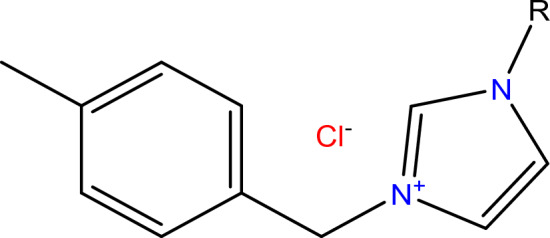
Structure of the prepared ILs (R_8_-IL, R_10_-IL, and R_12_-IL). Where, R = C_8_H_17_ for 3-(4-methylbenzyl)-1-octyl-1-H-imidazolium chloride named as R_8_-IL; R = C_10_H_21_ for 3-(4-methylbenzyl)-1-decyl-1-H-imidazolium chloride named as R_10_-IL; R = C_12_H_25_ for 3-(4-methylbenzyl)-1-dodecyl-1-H-imidazolium chloride named as R_12_-IL.

Three different ionic liquids (ILs) were prepared according to the method described elsewhere, as shown in Fig. 1,^30^. The synthesis method, in brief, was conducted by refluxing an equivalent amount of 1-(chloromethyl)-4-methylbenzene and 1-alkyl imidazole derivatives containing different alkyl chains for 6 h at 80 °C to produce 3-(4-methylbenzyl)-1-octyl-1-H-imidazolium chloride, 3-(4-methylbenzyl)-1-Decyl-1-H-imidazolium chloride, and 3-(4-methylbenzyl)-1-dodecyl-1-H-imidazolium chloride. The produced ILs named as R_8_-IL, R_10_-IL, and R_12_-IL, respectively showed a good solubility in a wide range of polar solvents^31^. Thin layer chromatography (TLC) method was used to verify the purity of the synthesized ILs using methylene chloride as the mobile phase.

#### NMR spectra of the prepared ILs

NMR spectra (^1^H-NMR and ^13^C-NMR) for the studied ILs; R_8_-IL, R_10_-IL, and R_12_-IL were investigated on a BRUKER NMR Spectrometer at frequency equal at 400 MHz and showed a similarity in the chemical shifts for the three ionic liquids. ^1^H-NMR spectroscopy were discussed in the previous work as shown before^30^. ^13^C-NMR (DMSO-d6) ppm for R_8_-IL, R_10_-IL, and R_12_-IL were expressed in Figs. S2, S3, & S4, respectively, and illustrated as shown below: For R_8_-IL: imidazolium carbons (135.44, 126.91, 123.18), pyridinium carbons (138.38, 131.38, 129.88), N–CH_2_ (62.37, 48.82), CH_2_–CH_2_ (30.03, 29.81), C–CH_3_ (22.51), CH_2_–CH_3_ (14.34). For R_10_-IL: imidazolium carbons (136.65, 128.57, 126.87), pyridinium carbons (139.98, 131.05, 129.73), N–CH_2_ (62.52, 49.20), CH_2_–CH_2_ (30.22, 29.39), C–CH_3_ (23.44), CH_2_–CH_3_ (14.26). For R_12_-IL: imidazolium carbons (136.7, 129.23, 128.26), pyridinium carbons (142.14, 132.92, 131.09), N–CH_2_ (62.37, 49.19), CH_2_–CH_2_ (31.97, 29.48), C–CH_3_ (22.58), CH_2_–CH_3_ (14.30).

### Solutions

Solutions of 1 M hydrochloric acid (37% analytical grade) containing various concentration of the prepared ILs of 20, 40, 60, 80, and 100 ppm were prepared using distilled water.

### Weight loss method

Carbon steel sheets used in weight loss have the same exposed surface area of 32.67 cm^2^. A set of emery papers were used to abrade the carbon steel sheets, then were cleaned based on the standard method G1-03/ASTM^32^. The specimens were precisely weighed before being placed in a 600 ml glass bottles with 500 ml of a preset concentration of HCl for 30, 60, 90, 120 and 150 min at temperatures ranges of 20, 35 and 50 °C. Various doses of the ILs were added to the acidic solutions to investigate their effect on the corrosion rate. The prepared acidic solutions were exposed to the atmosphere. After that they were removed at specific periods, cleaned, dried, and precisely weighed. A triple of each experiment was performed. Three sheets of carbon steel were weighed and the average was taken.

### Electrochemical measurements

A Potentiostat/Galvanostat (GAMRY 30000) connected to a computer with analysis software model (Gamry–Echem–Analyst) was used to output EIS and Tafel curves. Three electrodes; carbon steel, saturated calomel and platinum are as working electrode, reference electrode and auxiliary electrode, respectively in the glass cell. The working carbon steel electrode of surface area 0.345 cm^2^ was prepared and introduced in 100 ml of 1 M HCl through 3 h in presence and absence of certain concentrations of the prepared series of ILs in order to constancy the electrochemical system in gravimetric method. The Tafel graphs were performed under scan rate equal to 1 mV/s in the potential range − 700 to − 200 mV/SCE. The Nyquist presentation in the EIS method was performed at frequencies ranging (0.1 Hz–100 kHz) with a corrosion potential and amplitude of 20 mV.

### Analysis of protective film by FT-IR spectroscopy

The analytical Fourier transforms infrared (NICOLET iS50 FT-IR) instrument was the main used apparatus to determine the coated film composition stayed on the surface of the used metal through 24 h of immersion and R_10_-IL inhibitor. The collected powder which resulted after drying and scratching the specimen surface was analyzed. Vacuum dried R_10_-IL was used in ILs FT-IR experiment. The solid sample was mixed with spectroscopic grade KBr and introducing as KBr disk to the cell using an infrared lamp to be scanned. All of the tested samples spectra were obtained in the range of 4000 to 400 cm^−1^ under the rate of 32 cm^−1^/min.

### SEM and EDX surface analyses

The carbon steel surface was analyzed using the scanning electron microscopy (SEM) and the energy dispersive analysis of X-ray technique (EDX). For scanning electron microscopy studies, surface of carbon steel specimens was examined after exposure to 1 M HCl in the absence and presence of a certain concentration of the studied compound. SEM model (PHILIPS/FEI) was used to examine the surface morphology of steel surface. EDX model (PHILIPS/FEI) was employed to investigate the elements on the steel surface. All of the tests had been carried out in the chemical warfare department belonging to the Ministry of Defense. All micrographs of the carbon steel coupons were carried out at a magnification of 1000.

## Results and discussions

### Weight loss method

#### Concentrations of ILs and inhibitory effect

**Figure 2:**
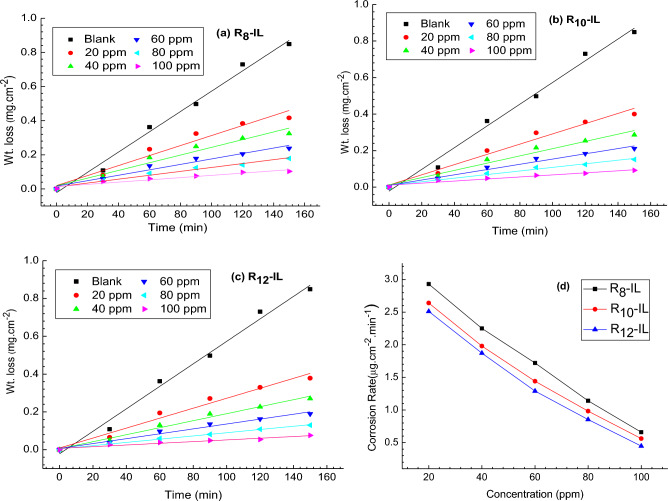
Influence of different ILs concentrations (**a**) R_8_-IL, (**b**) R_10_-IL and (**c**) R_12_-IL on carbon steel corrosion rate in 1 M HCl; (**d**) corrosion rate as a function of ILs concentration.

Weight loss-time curves of the carbon steel samples before and after the addition of R_8_-IL, R_10_-IL and R_12_-IL in 1 M HCl are shown in Fig. 2. The curves demonstrate that when the ILs concentrations in the acidic solution increase, the weight losses values (mg cm^−2^) of the carbon steel decrease, which enhances the inhibitor efficacy to impede corrosion. It is indicated that the formation of complex between ILs-donating species and carbon steel surface resulting the dissolution hindrance. The corrosion rate can be estimated using Eq. (1)^33^.

1 $$ {\text{C}} \cdot {\text{R}} = \Delta {\text{W}}/{\text{At}} $$

where ΔW represents the weight loss (mg), A is the area of the sample (cm^2^), while t is the time (minutes). The inhibition efficiency; IE% of the utilized ILs on the carbon steel surface at different studied concentrations was determined related to the calculated rate of corrosion as shown in Eq. (2).

2 $$ {\text{\% IE}} =\uptheta \times 100 = \left[ {\left( {{\text{W}}_{1} - {\text{W}}_{2} } \right)/{\text{W}}_{1} } \right] \times 100 $$

where W_1_ and W_2_ represent the weight loss of carbon steel without and with ILs as inhibitor, respectively. The inhibition efficiency data regarding the weight loss study in different concentrations of ILs in acidic medium (1 M HCl) are recorded in Table 1. The optimum dosage of the synthesized ILs was detected to determine the lowest rate of corrosion would be achieved at 100 ppm of all the ILs in 1 M HCl.

**Table 1:** Effect of various concentrations of the prepared ILs on the weight loss of the carbon steel samples in acidic medium (1 M HCl) at various temperatures.

Cpd	Conc., ppm	Temperature, °C								
		20			35			50		
		CR, µg. cm^−2^ min^−1^	θ	IE, %	CR, µg cm^−2^ min^−1^	θ	IE, %	CR, µg cm^−2^ min^−1^	θ	IE, %
Blank	0	5.95	–	–	6.54	–	–	7	–	–
R_8_-IL	20	2.93	0.508	50.8	3.43	0.476	47.6	4	0.429	42.9
	40	2.25	0.622	62.2	2.6	0.602	60.2	3.16	0.549	54.9
	60	1.59	0.711	71.1	2.03	0.689	68.9	2.39	0.659	65.9
	80	1.14	0.808	80.8	1.5	0.771	77.1	1.83	0.739	73.9
	100	0.66	0.889	88.9	1.03	0.843	84.3	1.4	0.80	80
R_10_-IL	20	2.8	0.556	55.6	3.34	0.489	48.9	3.93	0.439	43.9
	40	1.98	0.667	66.7	2.46	0.624	62.4	2.92	0.583	58.3
	60	1.44	0.758	75.8	1.87	0.714	71.4	2.24	0.68	68
	80	0.98	0.835	83.5	1.34	0.795	79.5	1.7	0.757	75.7
	100	0.56	0.906	90.6	0.90	0.862	86.2	1.31	0.813	81.3
R_12_-IL	20	2.63	0.578	57.8	3.2	0.511	51.1	3.77	0.461	46.1
	40	1.85	0.686	68.6	2.28	0.651	65.1	2.81	0.599	59.9
	60	1.29	0.783	78.3	1.7	0.74	74	2.04	0.709	70.9
	80	0.85	0.856	85.6	1.2	0.816	81.6	1.56	0.777	77.7
	100	0.44	0.925	92.5	0.818	0.875	87.5	1.2	0.829	82.9

#### Temperatures and inhibitory effect

**Figure 3:**
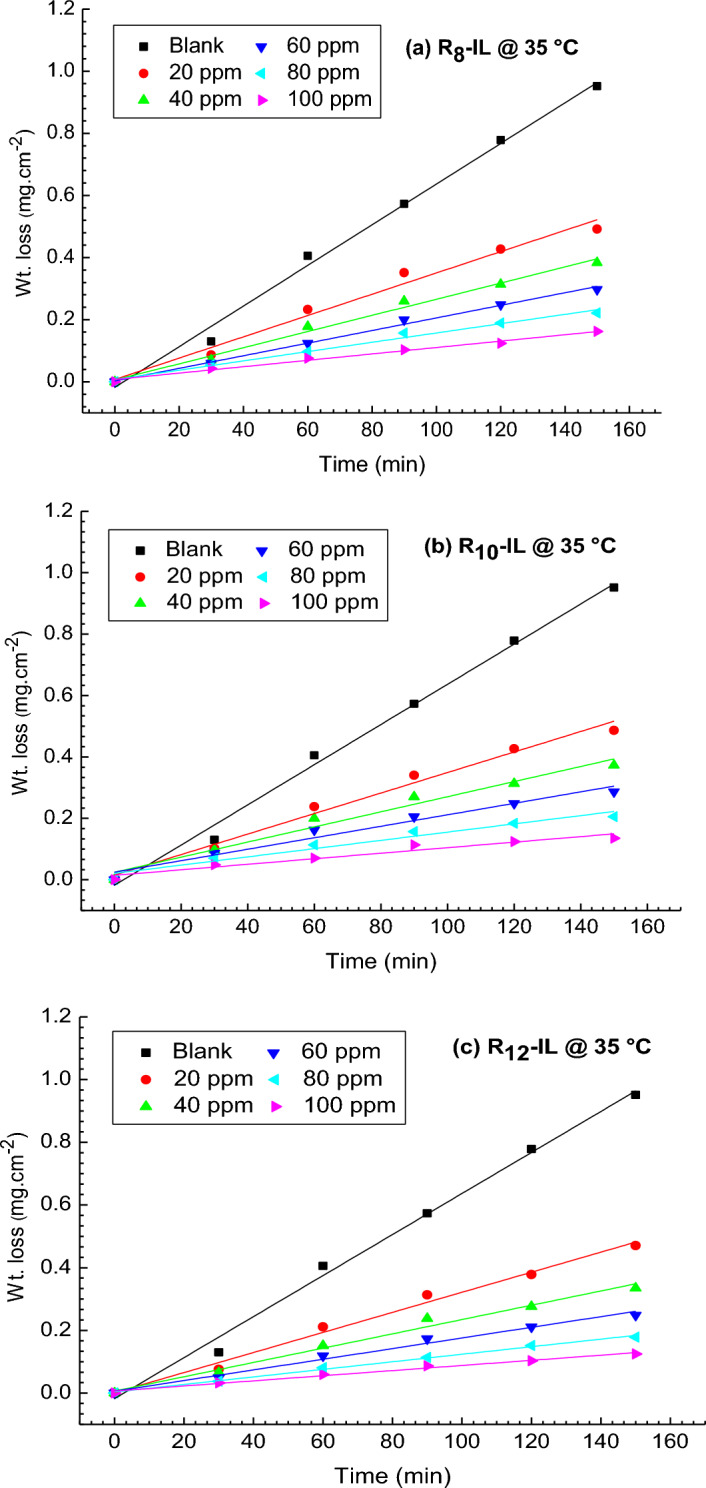
Influence of temperatures (35 and 50 °C) with the addition of various concentrations of (**a**) R_8_-IL, (**b**) R_10_-IL and (**c**) R_12_-IL in 1 M HCl on the weight losses of the carbon steel samples.

The corrosion rate of the synthesized ILs was examined at various temperature degrees to determine the created protected film stability. The effect of temperatures (35 and 50 °C) on the weight losses, and subsequently on the rate of corrosion of the carbon steel in the absence and presence of R_8_-IL, R_10_-IL and R_12_-IL, in the same concentration of acidic medium (1 M HCl) are shown in Fig. 3 (a, b, and c, respectively). Temperature significantly influences the rate that metal corrosion occurs. In an acidic solution, because the hydrogen evolution overpotential drops (hydrogen depolarization), the rate of corrosion increases exponentially with temperature^34^. From the investigation, the corrosion rate increases by increasing in temperature and the corrosion inhibition of the carbon steel surface eventually loses its efficacy. The data were calculated and presented in Table 1.

**Figure 4:**
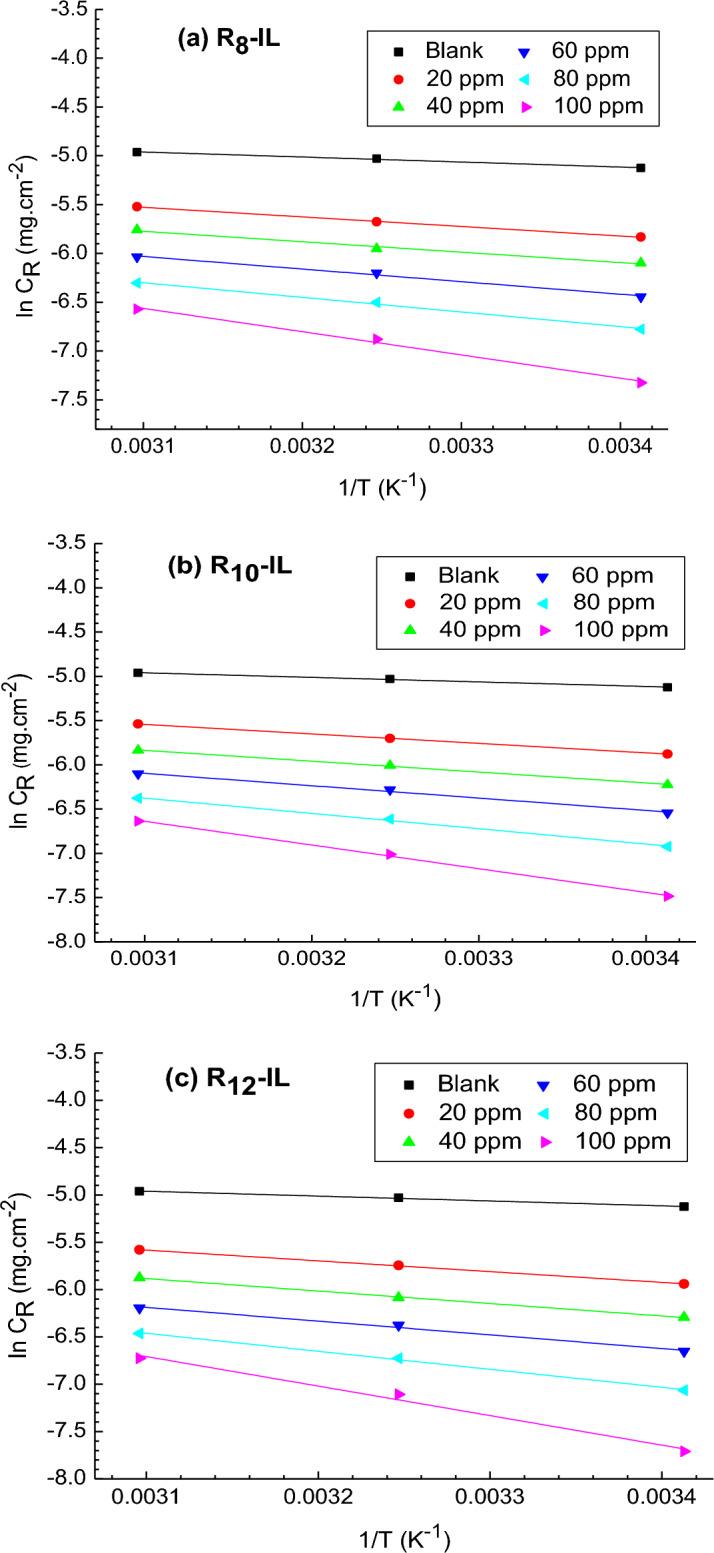
Arrhenius plot for the immersed carbon steel in 1 M HCl without and with different concentrations of (**a**) R_8_-IL, (**b**) R_10_-IL and (**c**) R_12_-IL.

Thermodynamic calculations and kinetic activation were demonstrated to explore both the corrosion inhibition mechanism and the characteristics of the protective film in the acidic solution. The apparent activation energy (E_a_) was calculated for R_8_-IL, R_10_-IL and R_12_-IL using Arrhenius equation (Eq. 3), and plotted in Fig. 4.

3 $$ C \cdot R = {\text{A}}\,\exp \left( {\frac{{E_{a} }}{RT}} \right) $$

In the previous equation, C.R represents the rate of corrosion, A, R, and T are the pre-exponential frequency factor (Arrhenius's factor), universal gas constant, and absolute temperature, respectively.

**Figure 5:**
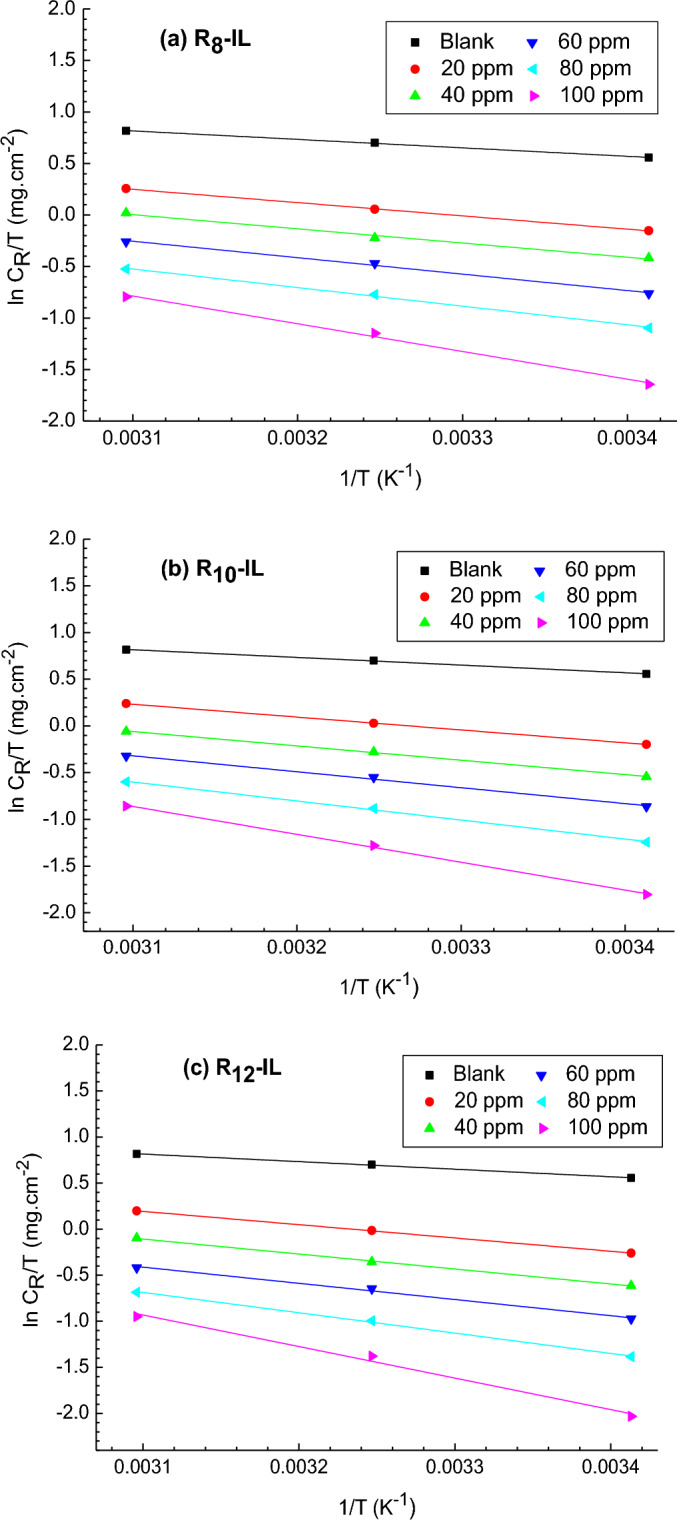
Transition state plot for the immersed carbon steel in 1 M HCl without and with different concentrations of (**a**) R_8_-IL, (**b**) R_10_-IL and (**c**) R_12_-IL.

Table 2 includes activation energy (E_a_) and the regulation factor (R^2^) for the untreated and treated carbon steel samples with the prepared ILs in 1 M HCl solution. Ea can be clarified by the modified mechanism of corrosion process due to the presence of blocking ILs compounds^35^. It is obvious that its values of the inhibited carbon steel is higher than that of the un-inhibited one, implying that greater energy barrier is needed to exhibit the corrosion reactions. Kinetic activation parameters have been evaluated using Fig. 5 and Eq. (4):

4 $$ C \cdot R = \frac{RT}{{N_{A} h}}\exp \left( {\frac{{\Delta S^{*} }}{R}} \right)\exp \left( {\frac{{\Delta H^{*} }}{RT}} \right) $$

where N_A_ is Avogadro’s number and h is the Planck’s constant, in addition to the entropy of activation (ΔS^*^) that was determined from the plot intercept, and the enthalpy of activation (ΔH^*^) that was determined from the plot slope. It is observed that ∆H^*^ has positive values which means the endothermic activation process. It is also seen that ∆H^*^ values increase by increasing the ILs concentrations in the same fashion as that for E_a_, indicating the role of the prepared ILs in increasing the formed energy barrier that diminishes the loss of the carbon steel in the acidic medium, allowing carbon steel to have a limited part in the corrosion process^36^. ∆S^*^ values were negative and increased with the increase of ILs concentration, i.e., the less negative values of ∆S^*^ reveals that the activated complex is less ordered by ILs where the reaction occurs on going from reactant to the activated complex, and the activated complex in the rate-determining step is associative. This is the result of the adsorption of the prepared ILs on the carbon steel surface, which replaces the adsorbed water^37^, suggesting that ILs play an effective role against the corrosion process. The reactions activation free energy, ΔG^*^, can be estimated at 20 °C by using the following equation:

5 $$ \Delta {\text{G}}^{*} = \Delta {\text{H}}^{*} - {\text{T}} \cdot \Delta {\text{S}}^{*} $$

**Table 2:** Carbon steel activation parameters in 1 M HCl solution before and after using R_8_-IL, R_10_-IL and R_12_-IL inhibitors.

Cpd	Conc., ppm	Thermodynamic and kinetic parameters			
		E_a_, kJ mol^−1^	∆H*, kJ mol^−1^	∆S*, kJ mol^−1^ K^−1^	∆G*, kJ mol^−1^
Blank	0	4.27	6.82279	− 0.169	56.49
R_8_-IL	20	8.16	10.71009	− 0.162	58.24
	40	8.88	11.42957	− 0.162	58.91
	60	10.71	13.26459	− 0.158	59.70
	80	12.43	14.98401	− 0.155	60.50
	100	19.8	22.35531	− 0.134	61.82
R_10_ − IL	20	8.87	11.44038	− 0.160	58.34
	40	10.2	12.74995	− 0.158	59.18
	60	11.6	14.16223	− 0.156	59.94
	80	14.4	16.92812	− 0.150	60.88
	100	22.25	24.80626	− 0.127	62.23
R_12_-IL	20	9.46	12.00047	− 0.158	58.48
	40	10.95	13.50358	− 0.156	59.35
	60	12.05	14.59926	− 0.155	60.19
	80	15.8	18.35421	− 0.146	61.21
	100	25.9	28.46865	− 116.938	62.73

By increasing the ILs concentration the free energy of activation increases, that attributed to the formation of unstable activated complex in the rate determining transition state.

### Adsorption isotherms

**Figure 6:**
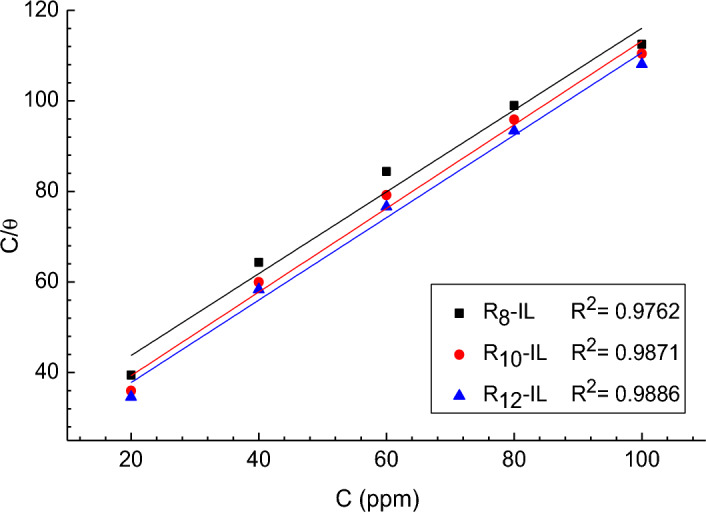
Langmuir isotherm of the studied ILs at 20 °C.

The adsorption process of the inhibitor is defined as the displacement reaction where the adsorbed water molecule is replaced by the inhibitor molecule on the metal surface^38^. In acidic solutions, the adsorption behavior of the inhibitor causes the transition of the metal/solution interface that is responsible for forming a protective film, and hence, the reactive metal surface is shielded from the acid solutions^39^. It is known that the inhibitor chemical structure, the nature, distribution of charge overall the inhibitor molecule and charged surface of the metal specifies the type of adsorption on the metal surface and the adsorption process. The best fit to the experimental data obtained from weight loss measurements were adapted to Langmuir adsorption Isotherm^40^. The correlation coefficient R^2^ of the Langmuir isotherm of the studied ILs was found to be in the range of 0.976–0.988 and the slopes are close to 1, as shown in Fig. 6. Table 3 contains the calculated values of the equilibrium constant of adsorption/desorption process (K_ads_) and the standard Gibbs free energy of adsorption (∆G°_ads_) according to Eqs. 6 and 7^41,42^. It is obvious that ∆G°_ads_ has negative values for the prepared ILs, which indicate the spontaneous adsorption of the ILs on the carbon steel surface^43^, and being between − 20 and − 40 kJ mol^−1^ shows an association of physisorption and chemisorption for the ILs on the carbon steel surface. In addition, the reported data revealed that increasing the alkyl chain length, increases the negative values of the negative free energy values.

6 $$ \frac{{C_{inh} }}{\uptheta } = \frac{1}{{{\text{K}}_{{{\text{ads}}}} }} + C_{inh} $$

7 $$ \Delta {\text{G}}^\circ_{{{\text{ads}}}} = - {\text{RT}}\,\ln \left( {10^{6} \,{\text{K}}_{{{\text{ads}}}} } \right) $$

where R is the universal gas cons. and T is temperature in Kelvin.

**Table 3:** Langmuir isotherm parameters for the studied ILs at 20 °C.

Cpd	R^2^	K_ads_, ppm^−1^	ΔG_ads_, kJ mol^−1^
R_8_-IL	0.9762	0.03893	− 26.17
R_10_-IL	0.9871	0.04803	− 26.7
R_12_-IL	0.9886	0.05109	− 26.85

### Potentiodynamic polarization

**Figure 7:**
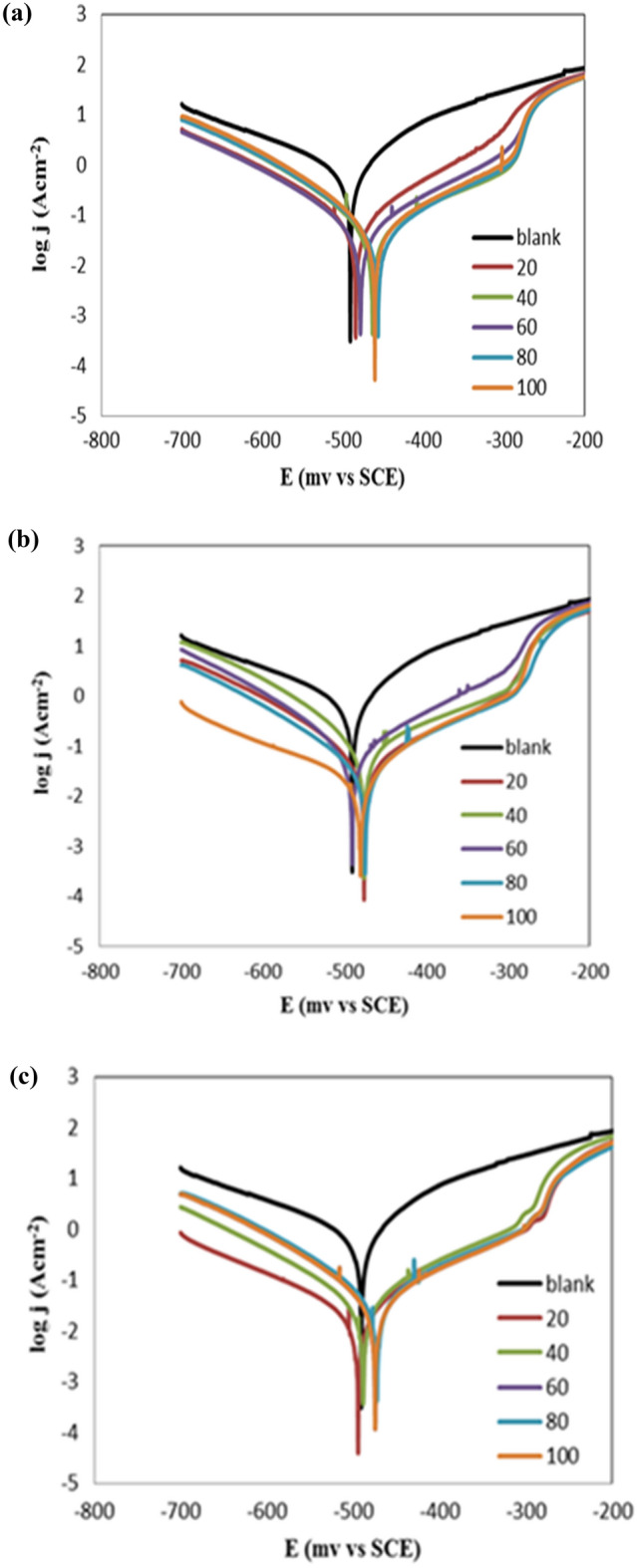
Potentiodynamic polarization of carbon steel (immersed in 1 M HCl) at temperature of 20 °C after adding different concentrations of (**a**) R_8_-IL, (**b**) R_10_-IL, and (**c**) R_12_-IL.

Tafel polarization data have been used to identify the kinetics and the electrochemical characteristics of the corrosion process. Figure 7 shows the relation between the current density (log I_corr_) and the corrosion potential (E, mV) versus the reference electrode (SCE) for the carbon steel electrode immersed in the acidic solution of 1 M HCl without and with different concentrations of the prepared ILs. According to the polarization curves, adding ILs induces the anodic/cathodic polarization curves to shift towards a noble direction, which shows a reduction in the rate of corrosion of carbon steel^44^. Moreover, changing the corrosion potential suggests that the prepared ILs can be considered as mixed type inhibitors^45^. Table 4 contains the values of the estimated electrochemical parameters like I_corr_, E_corr_, βa, and βc. It is obvious that the current density is significantly reduced by increasing both inhibitor doses and the substituted alkyl chain length. The surface coverage, θ, has been calculated from [1 − (I_corr_/I^o^_corr_)] and the inhibition efficiency percentage, η_T_ (%) by Tafel extrapolation are calculated according to Eq. (8)^46^. According to the mentioned parameters in Table 4, the R_12_-IL can be considered as the most efficient corrosion inhibitor. This could be attributed to the increase in substituted alkyl chain length, which in turn increase the surface area per molecule of each ionic liquid and grant it the ability to conceal the metal surface from the acidic medium.

8 $$\upeta _{{\text{T}}} \,\left( \% \right) = 1 - \left( {{\text{I}}_{{{\text{corr}}}} /{\text{I}}^{{\text{o}}}_{{{\text{corr}}}} } \right) \times 100 $$

where η_T_ (%) is the inhibition efficiency percentage, I^o^_corr_ is the current density without adding the inhibitor, and I_corr_ is the current density after adding the inhibitor.

**Table 4:** Data from potentiodynamic polarization of carbon steel (immersed in 1 M HCl) in different concentrations of ILs.

Cpd	Conc., ppm	E_corr_, mV	I_corr_, µA	β_a_, mV dec^−1^	β_c_, mV dec^−1^	η_T,_ %
Blank	0	− 490.6	1047.4 (± 0.91)	107.3 (± 0.35)	− 192.8 (± 1.27)	–
R_8_-IL	20	− 484.1	81.6 (± 0.23)	107.5 (± 0.3)	− 111.3 (± 0.99)	92.21
	40	− 463	51.19 (± 0.9)	138.9 (± 0.25)	− 90.3 (± 0.49)	95.11
	60	− 478	47.1 (± 0.9)	110.5 (± 0.5)	− 94.4 (± 0.78)	95.50
	80	− 456.3	43.7 (± 1.2)	115.1 (± 0.8)	− 89.2 (± 0.56)	95.83
	100	− 460.2	40.7 (± 0.75)	99.4 (± 0.5)	− 73.2 (± 1.13)	96.11
R_10_-IL	20	− 476.3	88.7 (± 1.05)	145.2 (± 0.3)	− 81.5 (± 0.7)	91.53
	40	490.9	73.26 (± 1.05)	110.7 (± 0.7)	− 87.8 (± 1.13)	93.01
	60	− 476.4	46 (± 1)	129.9 (± 0.8)	− 88.8 (± 1.13)	95.61
	80	− 474.8	39.99 (± 1.1)	119.8 (± 1.15)	− 102.1 (± 1.27)	96.18
	100	− 480.9	32.43 (± 0.5)	109.1 (± 1)	− 214.3 (± 0.49)	96.90
R_12_-IL	20	− 472.5	48.79 (± 0.8)	121.9 (± 1)	− 98.4 (± 0.78)	95.34
	40	− 485.5	49.47 (± 0.5)	127.3 (± 1.3)	− 108.6 (± 0.85)	95.28
	60	− 474.7	41.27 (± 0.8)	123 (± 1.05)	− 95.7 (± 0.99)	96.06
	80	− 488.3	36.29 (± 0.7)	104.1 (± 1)	− 107.4 (± 0.63)	96.54
	100	− 494.4	29.78 (± 0.6)	118.9 (± 0.35)	− 156 (± 1.13)	97.16

**Figure 8:**
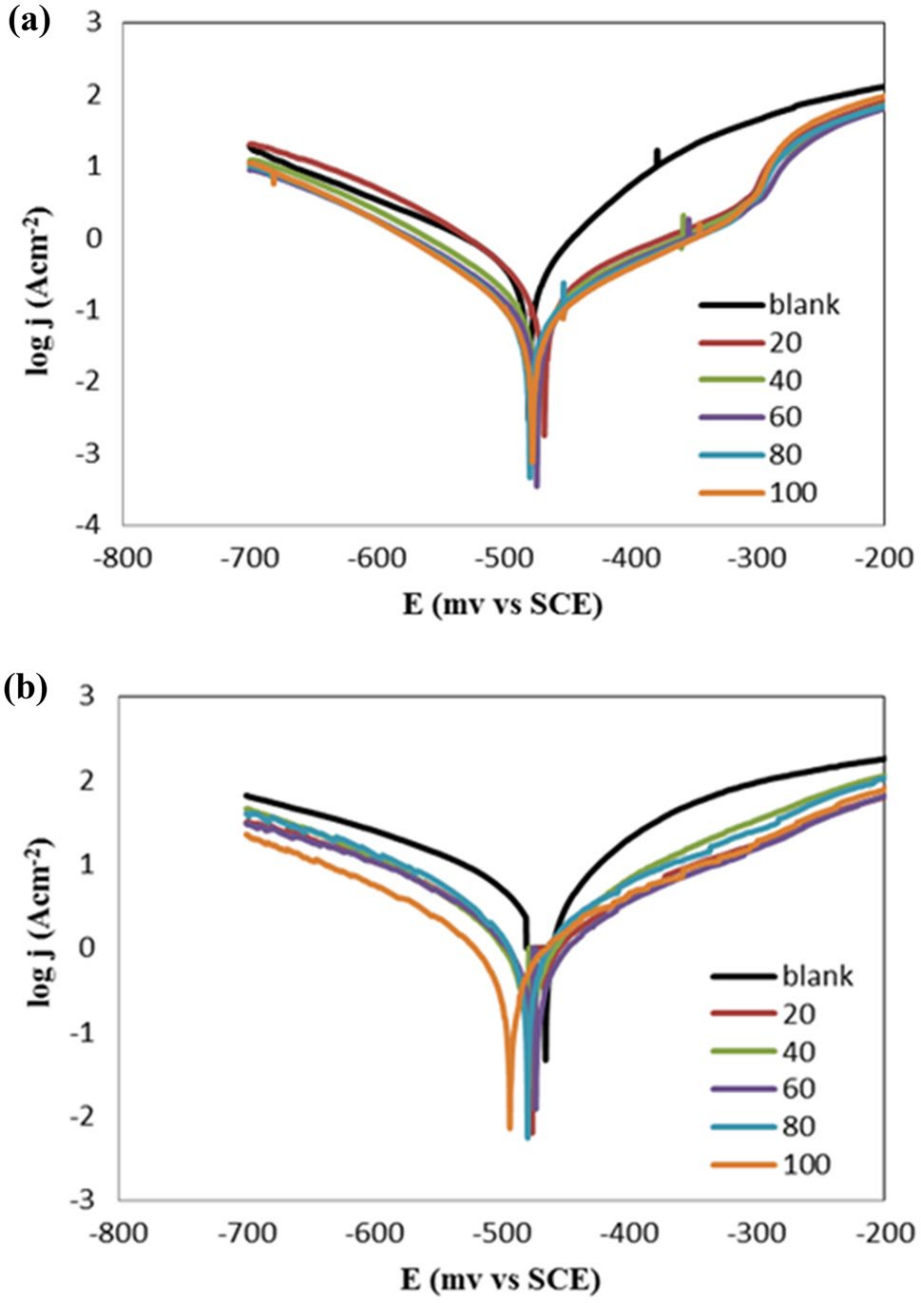
Influence of temperatures on potentiodynamic polarization curves for carbon steel (immersed in 1 M HCl) before and after adding different concentrations of R_12_-IL. (**a**) at 35 °C and (**b**) at 50 °C.

Additionally, the anticorrosive behavior of R_12_-IL was performed as a function of temperature at 35 °C and 50 °C and is shown in Fig. 8. The results showed that rising temperatures from 20 to 50 °C, significantly increase the corrosion current density and thus corrosion rate values. The influence of the temperature on η_T_ (%) is shown in Table 5. It was cleared that the inhibition efficacy value reduced from 97.16 to 77.27% with the temperature increase from 20 to 50 °C.

**Table 5:** Data from polarization of carbon steel immersed in 1 M HCl before and after adding different concentrations of R_12_-IL at 35 and 50 °C.

Temperature, °C	Conc., ppm	E_corr_, mV	I_corr_, µA	β_a_, mV dec^−1^	β_c_, mV dec^−1^	η_T,_ %
35 °C	0	− 480	577.7	80.3	− 151.8	–
	20	− 467.8	167.5	117.5	− 78.5	71.01
	40	− 473.6	130.1	126.7	− 97.4	77.48
	60	− 473.7	95.1	109.3	− 96.5	83.54
	80	− 479.1	90.4	119.5	− 91.1	84.35
	100	− 477	83.2	117.5	− 91.4	85.60
50 °C	0	− 465.9	4282.5	141.5	− 258.6	–
	20	− 475.9	2306.3	200.9	− 184.9	46.15
	40	− 478.3	2115.2	145.2	− 158.5	50.61
	60	− 473.5	1422.7	167.7	− 140.1	66.78
	80	− 479.4	1140	110.2	− 96.3	73.38
	100	− 493.2	973.6	155.1	− 139.9	77.27

### Electrochemical impedance (EIS)

**Figure 9:**
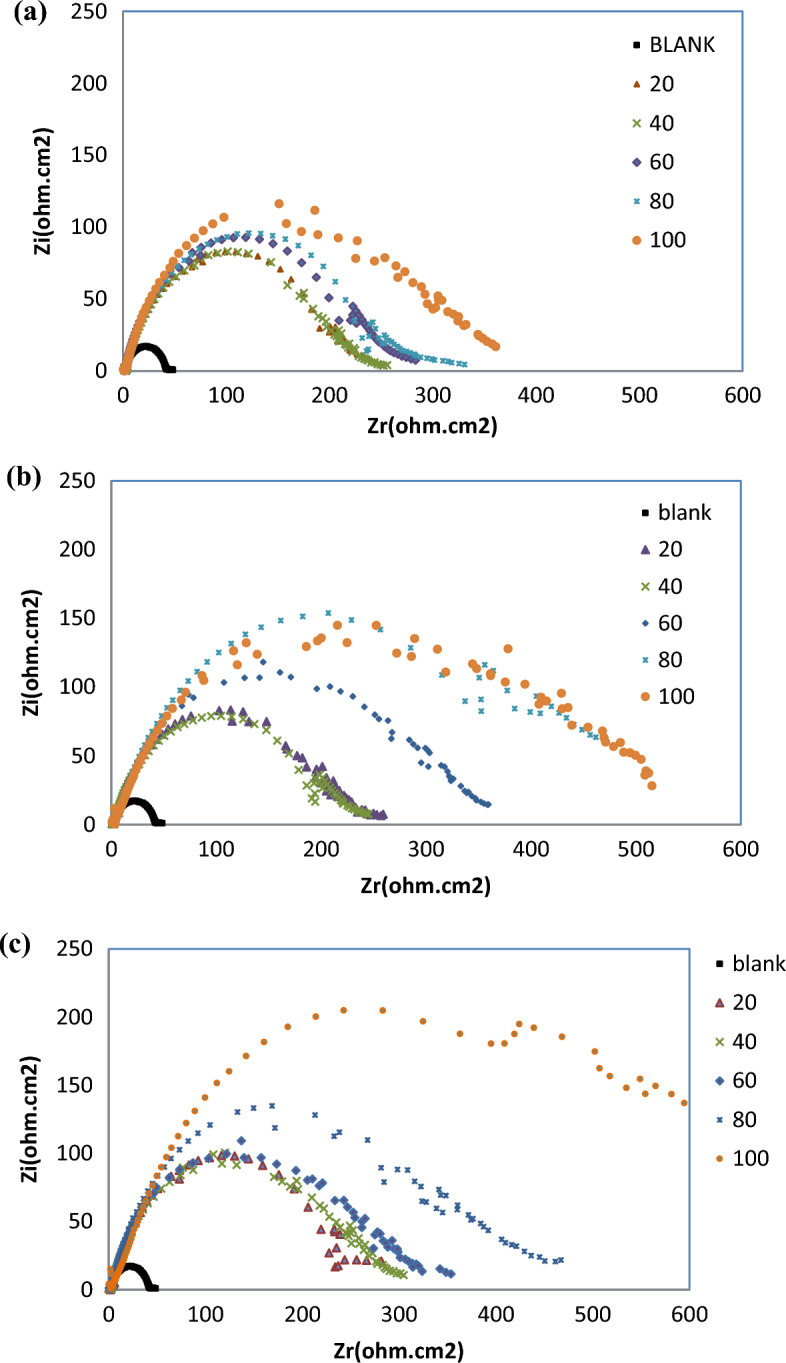
Nyquist plots of carbon steel specimen (immersed in 1 M HCl) before and after using different concentrations of ILs at 20 °C; (**a**) R_8_-IL, (**b**) R_10_-IL and (**c**) R_12_-IL.

**Figure 10:**
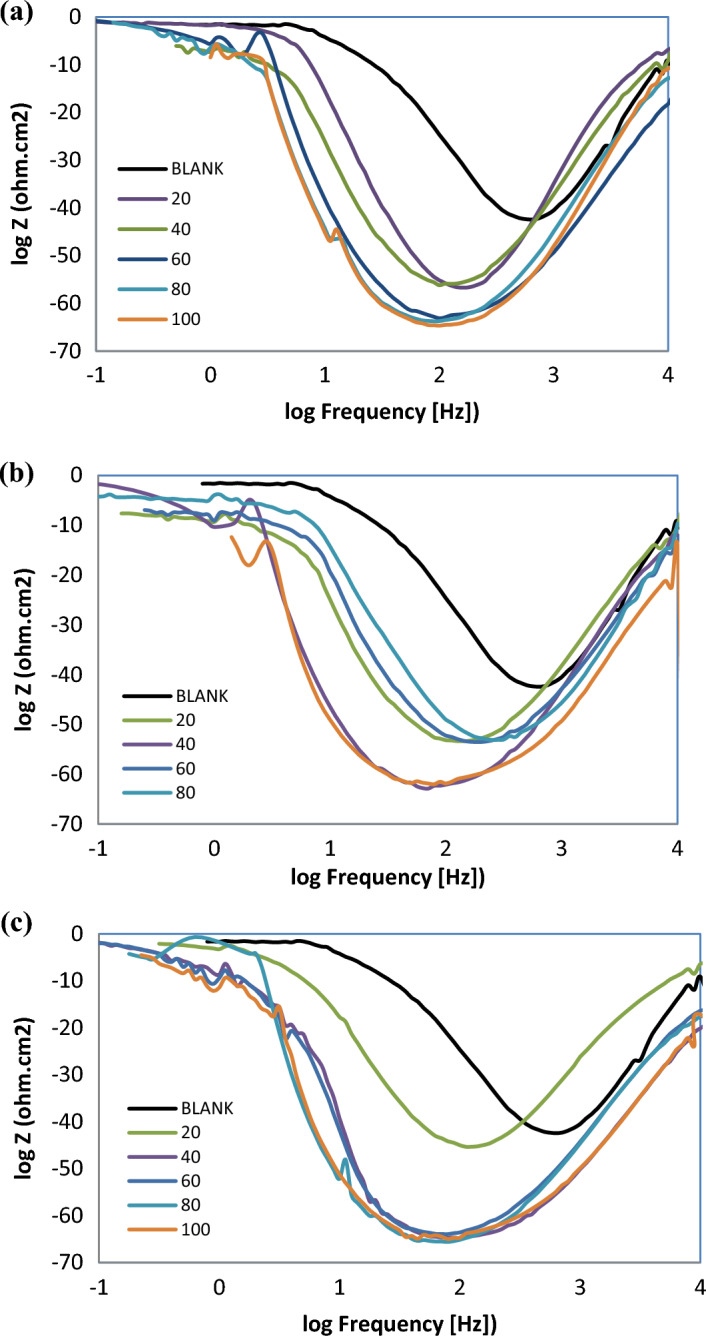
Bode plots for carbon steel specimen (immersed in 1 M HCl) before and after using different concentrations of ILs at 20 °C; (**a**) R_8_-IL, (**b**) R_10_-IL and (**c**) R_12_-IL.

**Figure 11:**
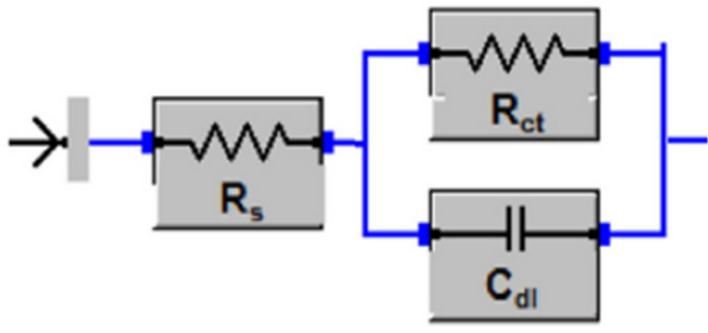
Equivalent circuit selected for interpreting the EIS data.

According to EIS analyses, the Nyquist plots displayed a resistance spectra of the carbon steel samples in 1 M HCl in the presence of various concentration of the prepared ILs; R_8_-IL, R_10_-IL and R_12_-IL (Fig. 9a–c, respectively). The equivalent circuit consists of the charge transfer resistance (R_ct_), the solution resistance (R_s_) and the double layer capacitance (C_dl_). It is shown that the resistance exhibited an expansion in size corresponding to variations in the amount of ionic liquids. By other words, the Nyquist plots exhibit a single depressed capacitive semicircle in the blank solution and the impedance modulus show the maximum by adding the inhibitors referring that the inhibitor concentration has an effective role in corrosion reaction. The results showed that all of the investigated ILs inhibitors enhance the resistance of the charge transfer (R_ct_) and decrease the capacitance (C_dl_). From the experimental findings, R_ct_ values increase until reaching its maximum effectiveness and C_dl_ values decrease which proves the adherence of inhibitor molecules on the metal surface forming a barrier film which hinders the diffusion of corrosive species towards the metal surface and may also act as a physical barrier to protect the metal surface from further corrosion. The solution's resistance (R_s_) remained consistently low regardless of the presence or absence of ionic liquids due to the high conductivity of all examined ionic liquids. It was noticed that the inclusion of ILs caused increasing of the overall resistance. Therefore, the presence of ILs in the HCl environment considerably positively altered the resistance responses of the carbon steel which explains the increase in the surface resistance by the increase of the ILs concentration. Based on the obtained results, the inhibition mechanism is controlled by charge transfer across the metal surface that has an inverse relation with the rate of corrosion^47^. Equation (9) is used to calculate η_EIS_ (%)^48^.

9 $$\upeta _{{{\text{EIS}}}} \,\left( \% \right) = 1 - \left( {{\text{R}}_{{{\text{ct0}}}} /{\text{R}}_{{{\text{ct}}}} } \right) \times 100 $$

where R_ct0_ is the charge transfer resistance without adding ILs and R_ct_ is the charge transfer after adding ILs inhibitors. It is clear that η_EIS_ (%) can be enhanced by increasing ILs concentration according to the data in Table 6. This increase is evidence that the adsorption of ILs on the metal surface can form a protective film and act as good inhibitors. The Bode plots of R_8_-IL, R_10_-IL and R_12_-IL are investigated and shown in (Fig. 10a–c, respectively). The results had shown that the presence of an adsorbed layer due to ILs inhibitor molecules increases the capacitive response of the interface, represents in the increased peak heights of the Bode curves. This was attributed to the formation of an electrochemical double layer with a capacitance which is consistent with the present findings. The electrochemical impedance confirmed the effectiveness of the prepared ILs as corrosion inhibitors for the carbon steel in acidic medium. Overall, elevating the concentrations of ionic liquids enhances the *η*_*EIS*_ % value up to critical concentrations. The highest *η*_*EIS*_ %, 98.33%, value was achieved with the ionic liquid, namely R_12_-IL, at 100 ppm. Figure 11 displays the best identical circuit for interpreting the EIS results. This circuit encompasses the components C_dl_ (double-layer capacitor), R_ct_ (charge transfer resistance), and Rs (solution resistance). The choice of this circuit could be attributed to provide the information needed for finding out the kinetic parameters and subsequently give more understanding about the reaction mechanism.

**Table 6:** Data parameters of EIS for the carbon steel sample immersed in 1 M HCl using different concentrations of the ILs.

Cpd	Conc., ppm	R_s_, Ω cm^2^	R_ct_, Ω cm^2^	C_dl_, µF cm^−2^	η_EIS_, %
Blank	0	1.1 (± 0.038)	9.33 (± 0.021)	107.68 (± 0.057)	–
R_8_-IL	20	1.78 (± 0.042)	214.86 (± 0.042)	104.62 (± 0.014)	95.66
	40	2.32 (± 0.036)	216.68 (± 0.056)	103.75 (± 0.035)	95.67
	60	2.27 (± 0.051)	232.39 (± 0.063)	121.78 (± 0.056)	95.99
	80	2.69 (± 0.035)	251.18 (± 0.056)	71.092 (± 0.0014)	96.29
	100	2.09 (± 0.055)	296.55 (± 0.035)	85.056 (± 0.005)	96.86
R_10_-IL	20	2.56 (± 0.05)	202.52 (± 0.014)	124.55 (± 0.035)	95.40
	40	1.79 (± 0.04)	214.1 (± 0.07)	117.81 (± 0.007)	95.64
	60	3.41 (± 0.038)	299.49 (± 0.063)	66.901 (± 0.022)	96.89
	80	1.8 (± 0.046)	450.7 (± 0.028)	55.966 (± 0.046)	97.93
	100	2.38 (± 0.053)	435.59 (± 0.063)	72.9 (± 0.042)	97.86
R_12_-IL	20	2.4 (± 0.047)	237.21 (± 0.007)	119.3 (± 0.056)	96.07
	40	1.8 (± 0.046)	270.57 (± 0.049)	65.998 (± 0.069)	96.55
	60	1.55 (± 0.05)	267.33 (± 0.021)	74.947 (± 0.033)	96.51
	80	1.76 (± 0.05)	359.64 (± 0.028)	78.694 (± 0.066)	97.41
	100	6.82 (± 0.041)	558.34 (± 0.028)	56.86 (± 0.042)	98.33

**Figure 12:**
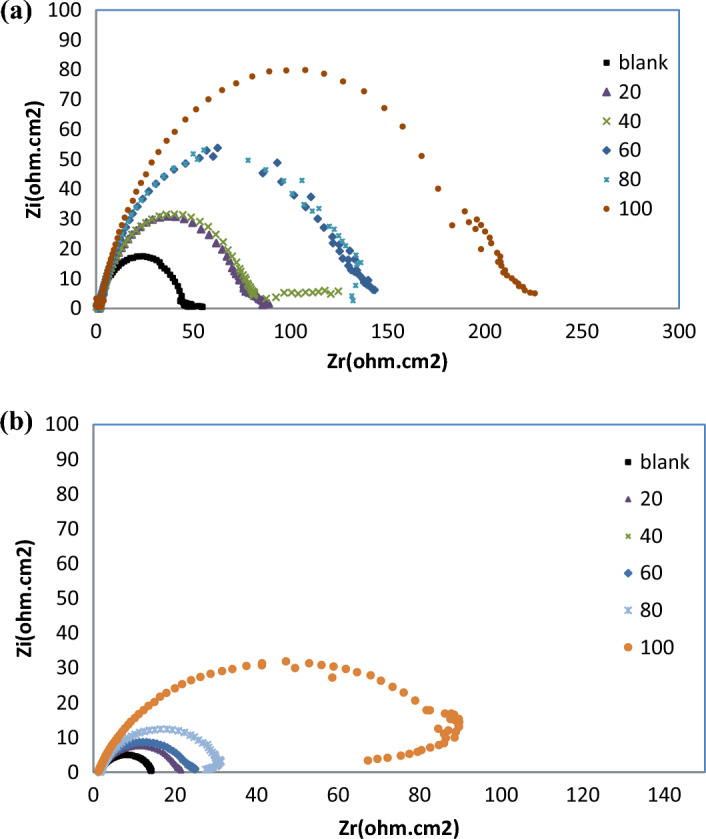
Influence of temperatures on Nyquist curves for carbon steel immersed in 1 M HCl containing various concentrations of R_12_-IL; (**a**) at 35 °C and (**b**) at 50 °C.

**Figure 13:**
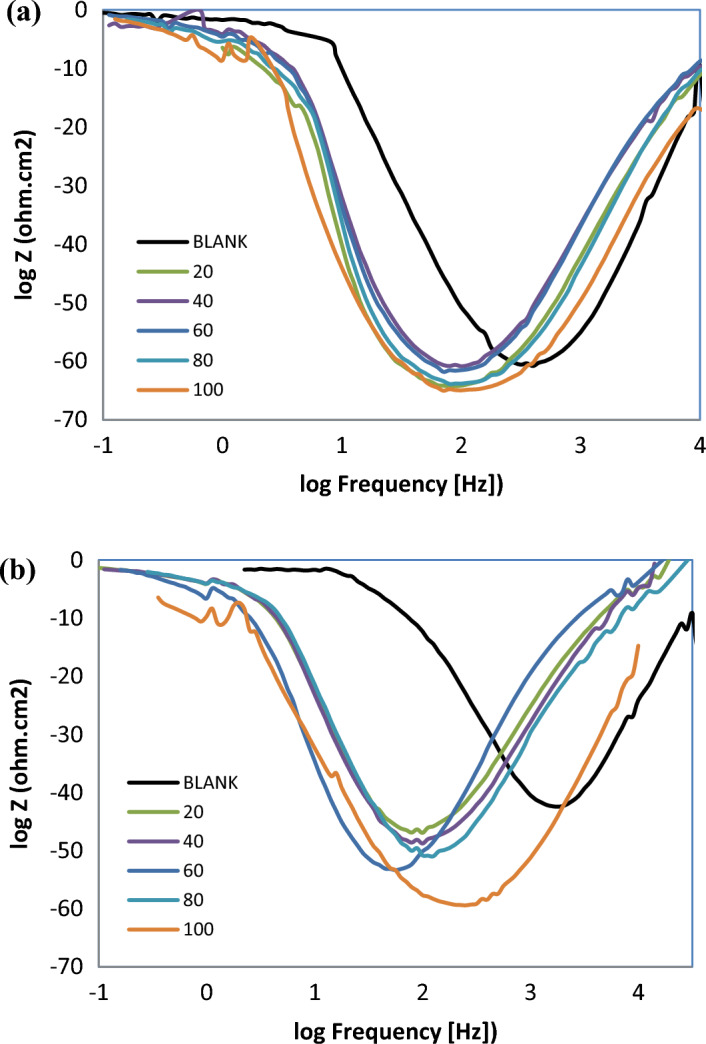
Influence of temperatures on Bode curves for carbon steel immersed in 1 M HCl containing various concentrations of R_12_-IL; (**a**) at 35 °C and (**b**) at 50 °C.

Effect of temperatures was also studied for R_12_-IL at 35 °C and 50 °C as shown in Fig. 12a,b, respectively, and the data were tabulated in Table 7. At the same temperatures ranges, by increasing the concentration of the ILs, both of the solution resistance (R_s_) and the charge transfer resistance (R_ct_) were increased which in turn represent in increasing the inhibition efficacy, while the double layer capacitance C_dl_ were decreased. This decline in the value of capacitance may be ascribed to the increased electrical double layer at the interface between the metal and the acidic solution and/or reduced local dielectric constant. Consequently, it may be assumed that the ionic liquid works as inhibitor by being adsorbed on the carbon steel. Moreover, the continued decline is brought by the steady replacement of the water molecules in the solution by the ionic liquid molecules, which lessens the degree of dissolving the carbon steel^49^. Based on the computed parameters, it was clear that the temperature affects negatively on the inhibition efficacy of the inhibitor. Simultaneously, in Bode plots at 35 °C and 50 °C, the phase angle increased with increasing concentration of the inhibitor until reaching its optimal level, Fig. 13a,b, respectively, as in R_12_-IL. All of these results suggest that the adsorbed inhibitor layer has a beneficial effect on the corrosion inhibition efficacy and are promising indicators of ILs effectiveness.

**Table 7:** Different parameters of EIS for the carbon steel in 1 M HCl with and without various concentrations of R_12_-IL at at 35 and 50 °C.

Temperature, °C	Conc., ppm	R_s_, Ω cm^2^	R_ct_, Ω cm^2^	C_dl_, µF cm^−2^	η_EIS_, %
35 °C	0	1.13	43.97	81.033	–
	20	1.57	76.35	185.79	42.41
	40	1.56	79.42	159.19	44.63
	60	1.971	83.19	151.97	47.14
	80	1.974	130.85	136.46	66.40
	100	2.12	197.88	101.25	77.78
50 °C	0	1.52	12.86	492.51	–
	20	1.28	20.16	443.84	36.20
	40	1.17	22.56	315.13	42.98
	60	1.38	22.79	392.73	43.54
	80	1.60	30.10	471.37	57.25
	100	964.53	92.83	152.79	86.14

### FT-IR spectra of film analysis

FT-IR analysis was performed for the protected film that has been found on the surface of the carbon steel samples after being immersed in the acidic medium containing 100 ppm of R_10_-IL inhibitor. The resultant spectrum was compared to the FT-IR spectrum of the R_10_-IL. The R_10_-IL constitutes of benzene unit, imidazole ring and alkyl chain^30^. The FT-IR spectrum of vacuum dried R_10_-IL is shown in Fig. S1a. The broad band that appeared around 3410 cm^−1^ is attributed to the hydrogen bonded H_2_O molecules^50^. The appeared band at around 3130 cm^−1^ is assigned to the vibrational motion of the C–H aromatic, while the aliphatic ones of the attached alkyl chain appeared between 2925 and 2854 cm^−1^^51^. The vibration peaks of the C–C inside the imidazolium ring and the aromatic C=C can be seen around 1561–1517 cm^−1^ and 1465 cm^−1^, respectively. Moreover, the stretching vibration motion of the C–N appeared at 1377 cm^−1^. On the other hand, Fig. S1b shows the resulted FT-IR spectrum of the coating that developed on the exposed surfaces of the carbon steel samples to the acidic medium with R_10_-IL. It is obvious that there are some exceptions to most of the peaks that found in the protective film with than in the by R_10_-IL. The peaks at 3130, 2956, and 2925 cm^−1^ that assigned to the aromatic C–H in the imidazolium ring, methyl, and methylene groups respectively, disappeared in the coating film spectrum. Moreover, disappearing of the vibration peak assigned to the C–N represented at 1377 cm^−1^, and the peaks around 1561 and 1517 cm^−1^ indicates the complication of iron with the IL or the formation of imidazole-iron salt. The appearance of a new peak at 462 cm^−1^ is attributed to the presence of γ-Fe_2_O_3_^52^. It was reported that the ILs can donates electrons to the carbon steel to attain its noble state of orbit and increase its stability. This delays the redox reactions and prevents the carbon steel surface from being attacked by corrosion.

### Surface analysis

**Figure 14:**
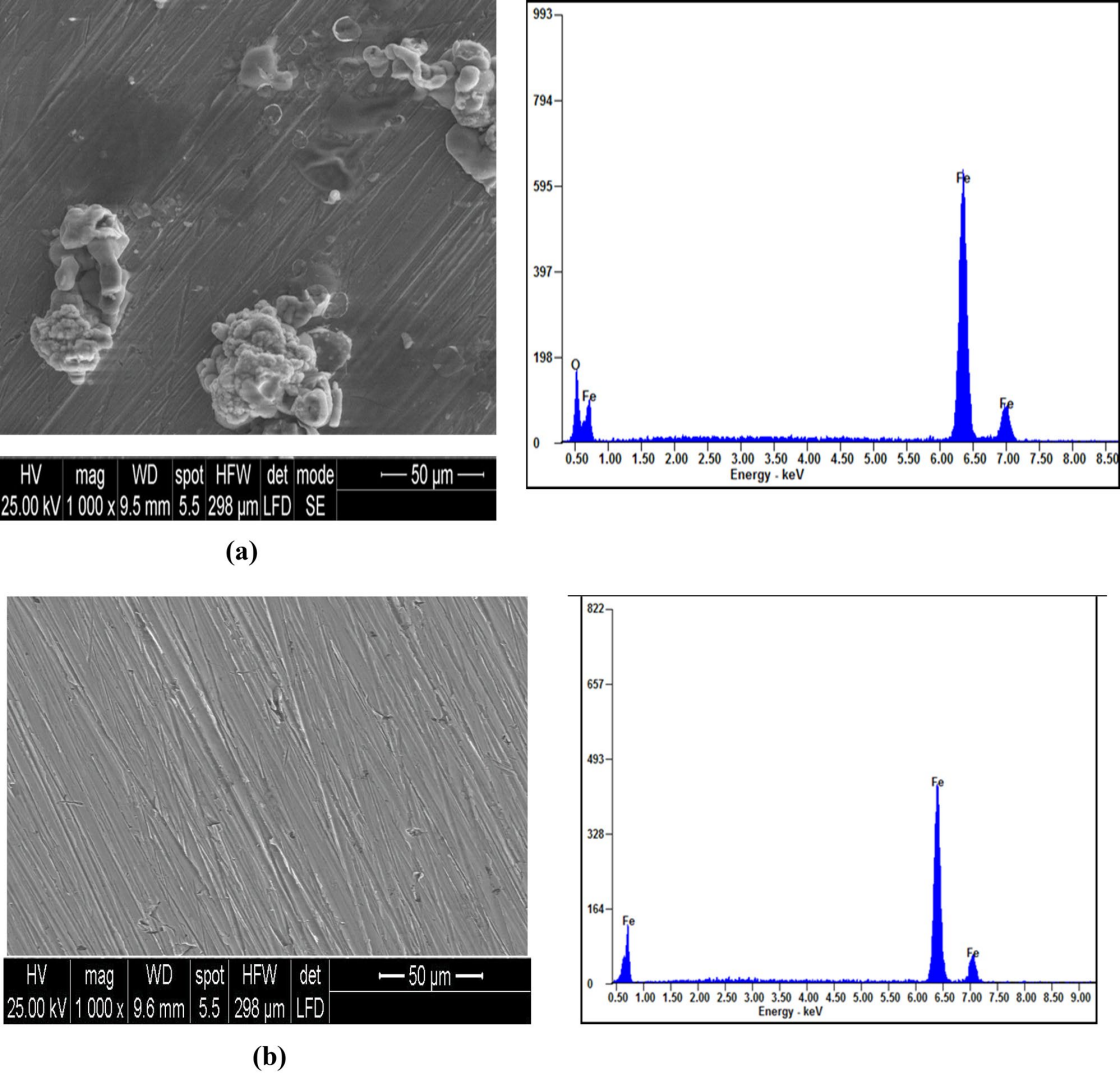
SEM and EDX images for the carbon steel surface: (**a**) after immersion in 1 M HCl, and (**b**) after immersion in the presence of 100 ppm of R_12_-IL, at 20 °C.

SEM image of the carbon steel coupon immersed in 1 M HCl environment through 4 h is shown in Fig. 14a. It is obvious that the coupon surface was badly damaged in the uninhibited solution and exhibits a number of pits and cracks. The carbon steel immersed through 4 h in the inhibited solution (1 M HCl + 100 ppm R_12_-IL) as shown in Fig. 14b, was noticeably improved with fewer cracks and pits as compared with the carbon steel surface in the uninhibited solution. This enhancement is the result of forming a prohibitive film on the carbon steel surface. Owing to the donating atoms that having unpaired electrons in addition to the way in which the π-electrons of double bonds inhibit carbon steel surface had excellent inhibitory efficacy.

The EDX spectrum of the uninhibited carbon steel coupon in Fig. 14a shows that it was severely weakened by external corrosion. By addition of 100 ppm of R_12_-IL inhibitor, the surface of the carbon steel was greatly improved due to formation of a protective film of the R_12_-IL molecules, as indicated by the decrease of iron band in Fig. 14b, showing that the protective film formed was strongly adherent to the surface, leading to a high degree of inhibition efficacy.

### Fukui indices

**Figure 15:**
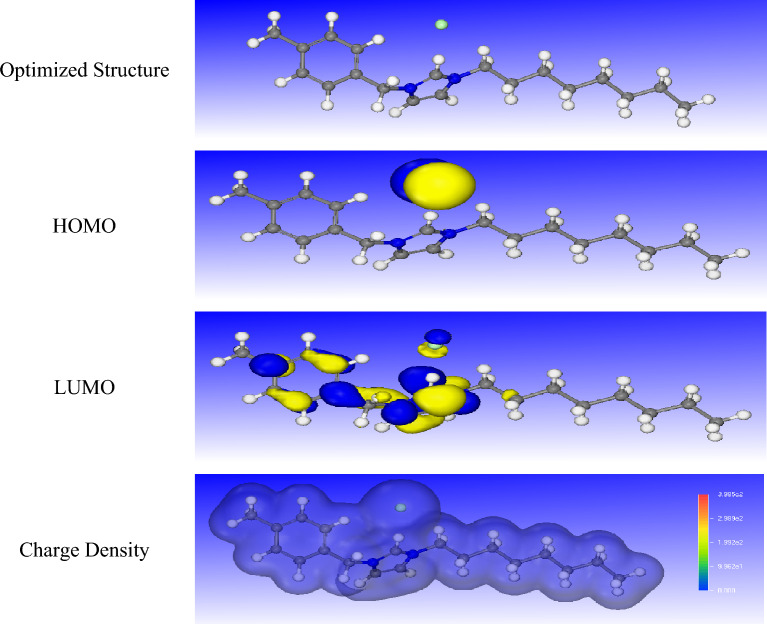
The optimized structures, HOMO, LUMO, and charge density of R_8_-IL.

**Figure 16:**
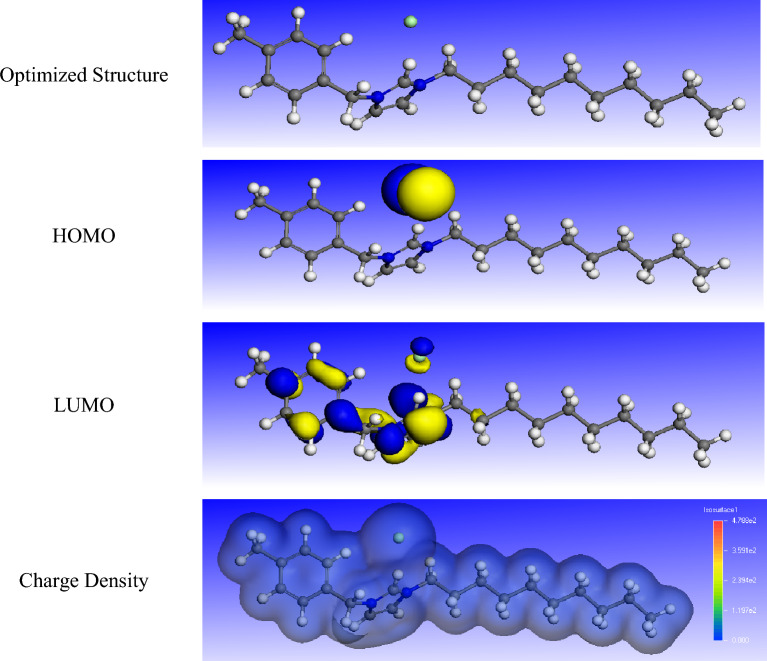
The optimized structures, HOMO, LUMO, and charge density of R_10_-IL.

**Figure 17:**
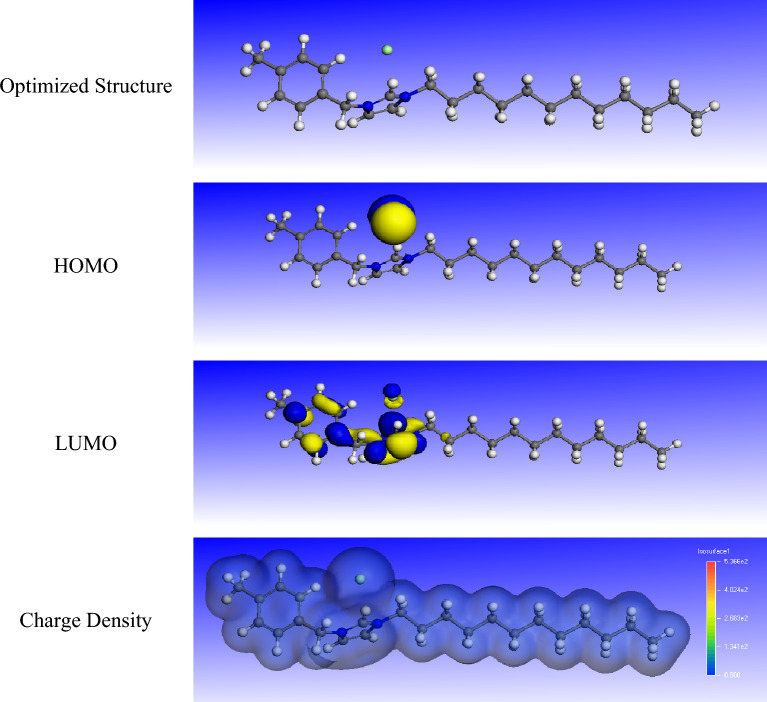
The optimized structures, HOMO, LUMO, and charge density of R_12_-IL.

The optimized structures, highest occupied molecular orbital (HOMO), lowest unoccupied molecular orbital (LUMO), and charge density of R_8_-IL, R_10_-IL, and R_12_-IL are presented in Figs. 15, 16 and 17, respectively. It is obvious that the electron density of the HOMO of the prepared ionic liquids is distributed over the chloride atom, while the LUMO is distributed close to the imidazole and the benzene rings. Fukui indices (f + and f-) of the prepared ionic liquids were calculated according to the electron charge distribution to determine the main electrophilic and nucleophilic sites on the ionic liquid molecules (Eqs. 10 and 11). They are very important to measure the local reactivity of the molecules.

10 $$ {\text{f}} - = {\text{N}} - {\text{N}} - 1 $$

11 $$ {\text{f}} + = {\text{N}} + 1 - {\text{N}} $$

The f + measures the reactivity of the molecule regarding the nucleophilic attack, while the f − indicates the reactivity of the molecule towards the electrophilic attack. Fukui indices of R_8_-IL, R_10_-IL, and R_12_-IL are listed in Tables S1, S2, & S3. It is clear that the most reactive sites in the R_8_-IL, R_10_-IL, and R12-IL as nucleophiles are Cl (51), Cl (57), and Cl (63), respectively, while for electrophiles, the most reactive sites are H (22), H (24), and H (26), respectively.

### Mechanism of inhibition

The inhibition mechanism of ILs may be explained as the adsorption of the ILs-donating species of ILs on the anodic sites of carbon steel, forming an adsorbed layer that restricts the surface from the solution and strengthen the protection of the metal surface. It was stated that imidazole-based IL has the ability to displace any adsorbed H_2_O molecules from the C-steel’s surface as commercial inhibitors^53^.

**Figure 18:**
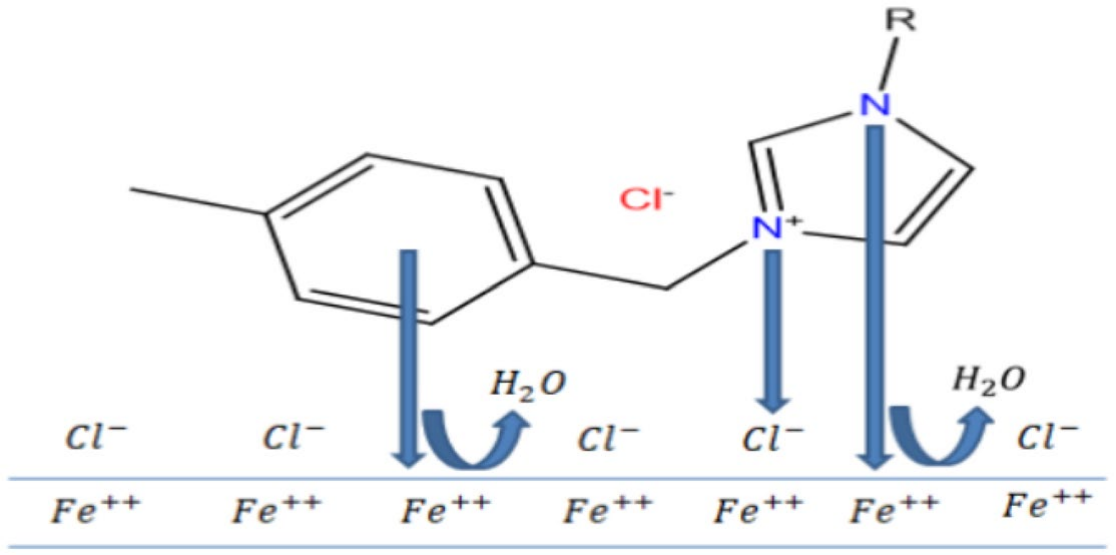
The proposed reaction mechanism that inhibits the corrosion of the carbon steel using ILs.

At first, physisorption process takes place as a result of the charged cations on the metal surfaces can interact electrostatically with the anions of the ILs. This interaction is mainly triggered by either dipole/dipole interactions or London force. In addition, physisorption may occurs via the interaction of anion species on the metal surfaces and the cationic parts of the imidazolium ILs. The presence of Vanderwaals forces between the ILs containing long chains of methylene groups, increases the stability of the coated film at the surface of the carbon steel. It is assumed that, at cathodic sites on the metal surface, the bulky IL’s cation can be adsorbed easily and replace the adsorbed H_2_O molecules (Fig. 18). Accordingly, the existence of inserted hetero-organic moieties, that provide more protection potential against the corrosion of steel, along with the entity of a long chain that increases the minimum area per molecule^54–56^. This interaction occurs through coordination bonds when ILs molecules adhere to create an restrictive layer at metal/solution interface. The presence of N=C–N moiety can easily provide active sites to be adsorbed on the metal surface^57^. Moreover, the adsorption process can be increased in the presence of multiple bonds.

In addition, the suggested chemical adsorption that occurs through a mechanism involves the interaction of π-electrons on the nitrogen atom of the imidazolium ILs and also π-electrons of the aromatic moieties and the vacant d-orbital of the carbon steel^58^, preventing it from dissolving in the aggressive medium.

It was also assumed that adsorption can occur via the cation species of the ILs that oriented parallel to the metal surface. The delocalization of the nitrogen atom of the imidazolium cation inside the ring produces a mild positive charge on the whole cation of the IL. Then, the cation of the IL intends to accept electrons from the carbon steel surface. This process has the ability to alter the polarity of the carbon steel surface, due to the formation of multilayer of the adsorbed species (N^+^ + Cl^−^/N^−^ + N^+^) on the surface^59^. On the other hand, the IL aromatic moieties donate the π electrons to the vacant d-orbitals in the surface of the carbon steel, which leads to the formation of donor–acceptor complexes^60,61^. The previous processes are responsible for an inter-electronic repulsion between the IL and the metal surface, which makes it easier for back bonding or retro-donation. Donation of electrons and back donation coupled strengthen one another, blocking the surface of the carbon steel, and subsequently preventing it from corrosion^62^, producing a stable chemical coating that reduces friction and wear reporting that imidazole-based IL are adsorbed on carbon steel surface by physical besides chemical adsorption process.

## Comparison with the previous works

In comparison with others publications, numerous inhibitors that have been tested and applied industrially, those that are nontoxic or of low toxicity, are now far more strategic than in the recent past such as Schiff base derivatives^63,64^ and ILs derivatives based as in this work. It was found that both of them are effective corrosion inhibitors which are attributed to their ability to spontaneously form a monolayer on the metal surface to be protected.

Compared with other publications, research in the field of green or eco-friendly corrosion inhibitors has been addressed toward the goal of using cost effective compounds with low or zero environmental impact.

When comparing the examined ILs with other inhibitors of steel in acidic media that have been reported, the comparative analysis introduces an evident that the ILs that evaluated had higher efficiency and stronger protective performance.

## Conclusions

The obtained results reveal that the investigated ILs have excellent performance in corrosion inhibition efficacy, η, at relatively economic doses in HCl acidic solution as eco-friendly inhibitors. From weight loss measurements, the inhibition efficacy of R_12_-IL was reached to be 92.5%, and 97.16% and 98.33% as measured by potentiodynamic polarization and electrochemical impedance spectroscopy, respectively and found to have the most effective inhibitor with optimum concentration 100 ppm at 293 K. The effect of temperature and immersion time were studied and thermodynamic parameters values of the interaction such as activation energy, enthalpy, entropy, and activation free energy were very satisfactory in the inhibition process. The inhibitive effect can be correlated to the adsorption of the ionic liquid species forming a protective film on the surface and that interpreted in the mode of adsorption that follows Langmuir isotherm and the parameters evaluation showed an association of physical and chemical adsorption.

The polarization results suggested that the ionic liquids function act as a mixed inhibitor as shown from shifting in current profile. The corresponding results of EIS measurements was compared to the results from Tafel polarization measurements at all studied temperatures and represented as the increase in charge-transfer resistance (R_ct_) and the decrease in double layer capacitance (C_dl_) and showed good compatibility.

FI-TR analysis of the layer formed on carbon steel showed that the R_10_-IL donating species form the adhered protective layer by complication with iron ions of carbon steel, as a representative sample of other ILs. The findings demonstrate the potency of R_12_-IL which gave better inhibition performance than the other ILs, followed by R_10_-IL then R_8_-IL, respectively.

## The importance of this work

Our work contribute to the field by the use of novel prepared ILs as carbon steel eco-friendly corrosion inhibitors in HCl solutions. The most important feature was the preparations of these ILs are environmentally safe, so, they have been extensively investigated recently and have more attention for the environmental restrictions.

In addition, ILs based Imidazolium salts have several advantages including that exhibit high inhibition performance against the corrosion of carbon steel in HCl solution and the inhibition efficacy reached to more than 98% and that been reported by electrochemical measurements. The results were applied using various techniques; weight loss as a chemical method, potentiodynamic polarization and electrochemical impedance spectroscopy as electrochemical measurements, in addition of protective film analysis by FT-IR spectroscopy.

The use of these ILs are cost-effective compared to other corrosion inhibitors and can be facilely prepared in the laboratory. The imidazolium ILs are effective corrosion inhibitors that can be used in harsh conditions. These properties make them crucial aspect for sustainable development.

Overall, the new ILs based Imidazolium salts as corrosion inhibitors for carbon steel in HCl solution were introduced to the field many additions as their high inhibition efficacy, non-toxicity, cost-effectiveness, and environmental friendliness and a promising candidate for industrial applications.

### Supplementary Information

Supplementary Information.

## Data Availability

The data that support the findings in the present study are available from the corresponding author upon request.
