# Supplementary material for: Evaluation of ionic liquids based imidazolium salts as an environmentally friendly corrosion inhibitors for carbon steel in HCl solutions
Source: Sci Rep. 2024 Jan 22;14:1889. doi: 10.1038/s41598-024-52174-5 (PMC10803315; doi:10.1038/s41598-024-52174-5)
Supplement: Supplementary file 1 — Supplementary Information. [file 41598_2024_52174_MOESM1_ESM.docx]

**Evaluation of Ionic Liquids based Imidazolium salts as an Environmentally Friendly Corrosion Inhibitors for Carbon Steel in HCl Solutions**

Raghda A. El-Nagar ^1^*, N.A. Khalil ^1^, Y. Atef ^2^, Maher I. Nessim ^1^, Alaa Ghanem ^3,4^*

^1^ Petroleum Testing Lab, Analysis & Evaluation Department, Egyptian Petroleum Research Institute, Nasr City, Cairo, 11727, Egypt.

^2^ Research Laboratory, General Organization for Export and Import Control (G.O.E.I.C), Cairo, Egypt.

^3^ PVT lab, Production Department, Egyptian Petroleum Research Institute, Nasr City, Cairo, 11727, Egypt.

^4^ PVT Services Center, Egyptian Petroleum Research Institute, Nasr City, Cairo, 11727, Egypt.

Corresponding Email: [alaa_ghanem2001@yahoo.com](mailto:alaa_ghanem2001@yahoo.com) & [alaa_ghanem2001@epri.sci.eg](mailto:alaa_ghanem2001@epri.sci.eg)

| 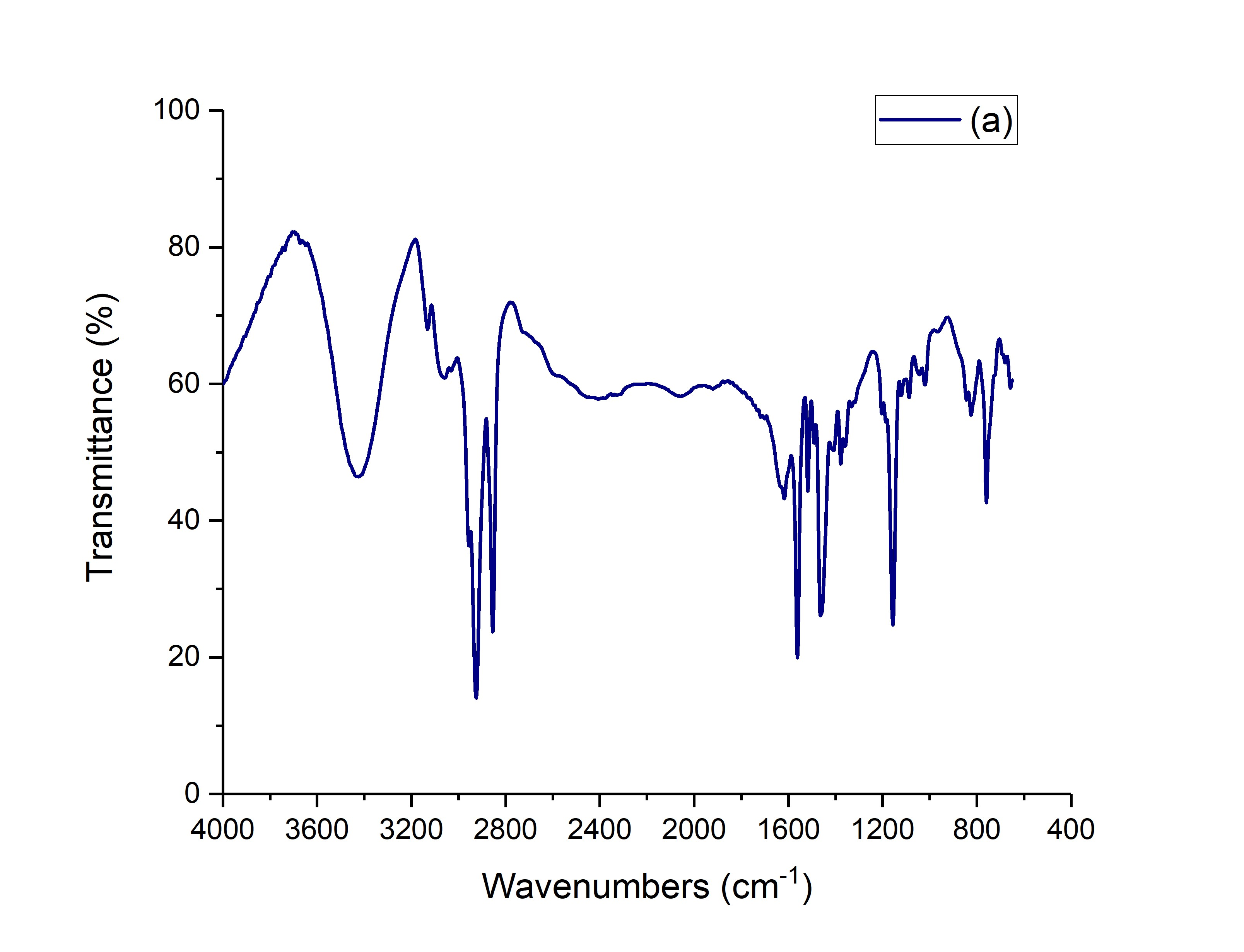 |
| --- |
| 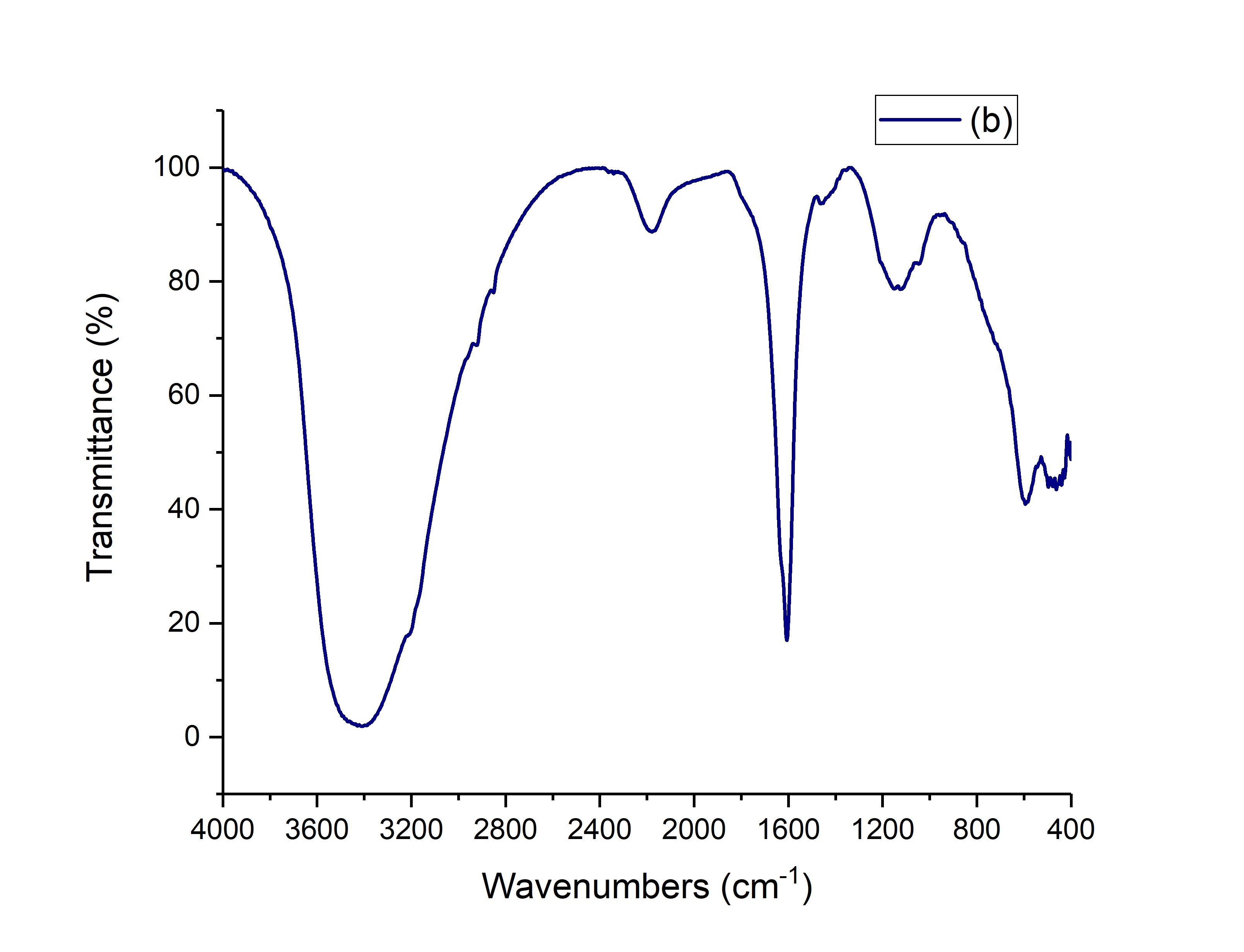 |
| Figure S1 FT-IR spectra of a) R_10_-IL and b) the formed preventive film on the carbon steel’s surface that immersed for two days in 1M HCl containing 100 ppm of R_10_-IL at 20 °C. |


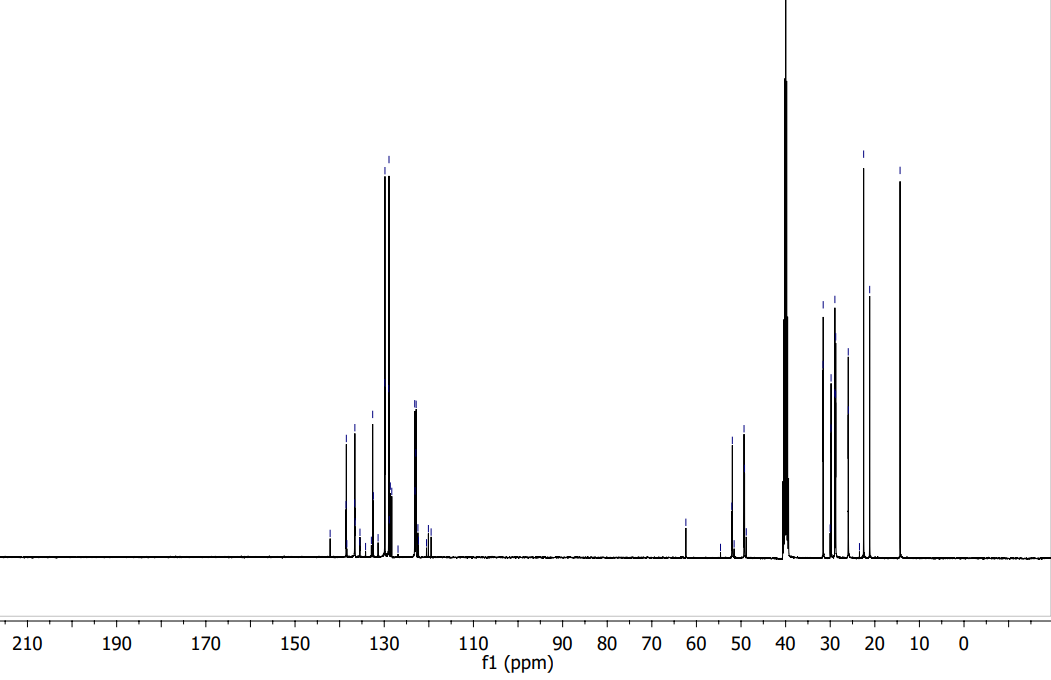


Figure S2 ^13^C-NMR values (ppm) of R_8_-IL


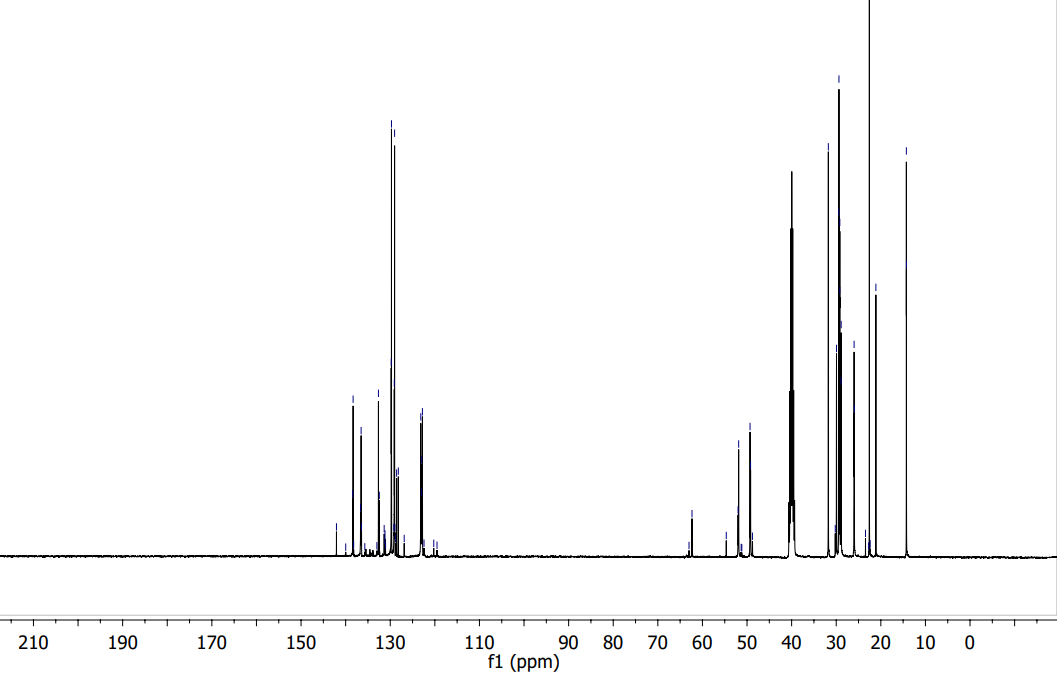


R_10_-IL

Figure S3 ^13^C-NMR values (ppm) of R_10_-IL


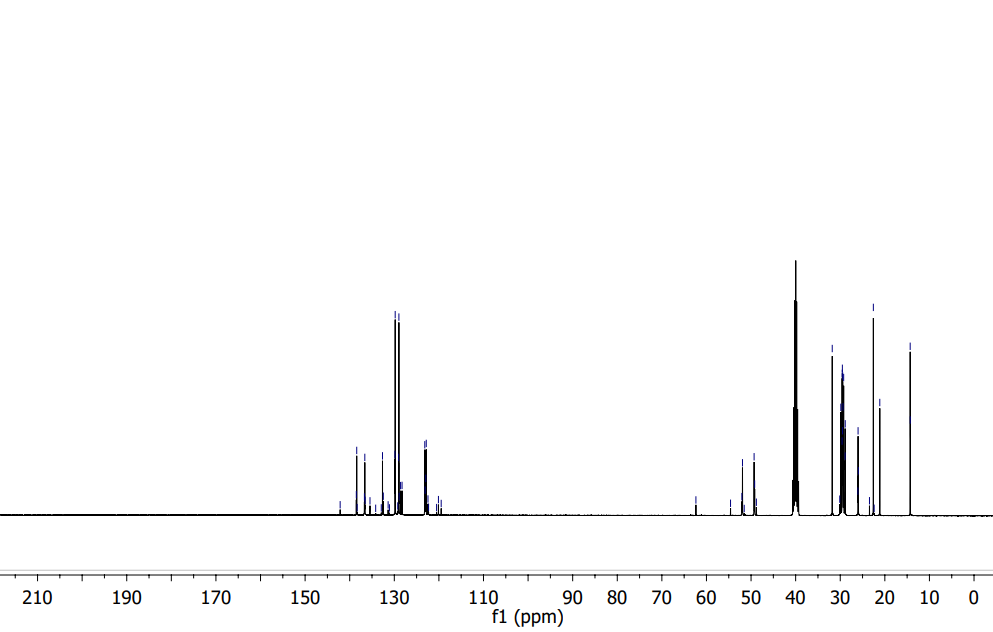

R_12_-IL

Figure S4 ^13^C-NMR values (ppm) of R_12_-IL

**Table S1** Fukui indices for the electrophilic and nucleophilic attacks on R_8_-IL

| Atom No. | N0 | N+1 | N-1 | f+ | f- |
| --- | --- | --- | --- | --- | --- |
| C ( 1) | 0.08 | 0.092 | 0.108 | 0.012 | -0.028 |
| N ( 2) | 0.01 | -0.218 | -0.216 | -0.228 | 0.226 |
| C ( 3) | 0.028 | -0.028 | -0.023 | -0.056 | 0.051 |
| C ( 4) | 0.028 | -0.03 | -0.025 | -0.058 | 0.053 |
| N ( 5) | 0.016 | -0.225 | -0.222 | -0.241 | 0.238 |
| C ( 6) | -0.016 | -0.191 | -0.195 | -0.175 | 0.179 |
| C ( 7) | -0.008 | -0.276 | -0.278 | -0.268 | 0.27 |
| C ( 8) | -0.004 | -0.271 | -0.272 | -0.267 | 0.268 |
| C ( 9) | -0.005 | -0.265 | -0.266 | -0.26 | 0.261 |
| C ( 10) | -0.003 | -0.262 | -0.262 | -0.259 | 0.259 |
| C ( 11) | -0.003 | -0.261 | -0.262 | -0.258 | 0.259 |
| C ( 12) | -0.003 | -0.258 | -0.258 | -0.255 | 0.255 |
| C ( 13) | -0.002 | -0.431 | -0.431 | -0.429 | 0.429 |
| C ( 14) | -0.019 | -0.261 | -0.265 | -0.242 | 0.246 |
| C ( 15) | 0.01 | 0.154 | 0.156 | 0.144 | -0.146 |
| C ( 16) | 0.012 | -0.189 | -0.186 | -0.201 | 0.198 |
| C ( 17) | 0.015 | -0.187 | -0.184 | -0.202 | 0.199 |
| C ( 18) | 0.018 | 0.172 | 0.176 | 0.154 | -0.158 |
| C ( 19) | 0.015 | -0.186 | -0.183 | -0.201 | 0.198 |
| C ( 20) | 0.011 | -0.214 | -0.212 | -0.225 | 0.223 |
| C ( 21) | -0.01 | -0.512 | -0.514 | -0.502 | 0.504 |
| H ( 22) | 0.01 | 0.252 | 0.254 | 0.242 | -0.244 |
| H ( 23) | 0.046 | 0.154 | 0.163 | 0.108 | -0.117 |
| H ( 24) | 0.05 | 0.152 | 0.162 | 0.102 | -0.112 |
| H ( 25) | 0.035 | 0.159 | 0.166 | 0.124 | -0.131 |
| H ( 26) | 0.006 | 0.219 | 0.22 | 0.213 | -0.214 |
| H ( 27) | 0.02 | 0.136 | 0.14 | 0.116 | -0.12 |
| H ( 28) | 0.004 | 0.158 | 0.158 | 0.154 | -0.154 |
| H ( 29) | 0.001 | 0.157 | 0.157 | 0.156 | -0.156 |
| H ( 30) | 0.015 | 0.135 | 0.138 | 0.12 | -0.123 |
| H ( 31) | 0.009 | 0.131 | 0.133 | 0.122 | -0.124 |
| H ( 32) | 0.004 | 0.139 | 0.139 | 0.135 | -0.135 |
| H ( 33) | 0.003 | 0.138 | 0.139 | 0.135 | -0.136 |
| H ( 34) | 0.007 | 0.132 | 0.133 | 0.125 | -0.126 |
| H ( 35) | 0.005 | 0.129 | 0.13 | 0.124 | -0.125 |
| H ( 36) | 0.003 | 0.133 | 0.133 | 0.13 | -0.13 |
| H ( 37) | 0.003 | 0.134 | 0.135 | 0.131 | -0.132 |
| H ( 38) | 0.005 | 0.132 | 0.133 | 0.127 | -0.128 |
| H ( 39) | 0.004 | 0.14 | 0.141 | 0.136 | -0.137 |
| H ( 40) | 0.003 | 0.142 | 0.142 | 0.139 | -0.139 |
| H ( 41) | 0.009 | 0.142 | 0.144 | 0.133 | -0.135 |
| H ( 42) | 0.043 | 0.164 | 0.172 | 0.121 | -0.129 |
| H ( 43) | 0.022 | 0.201 | 0.205 | 0.179 | -0.183 |
| H ( 44) | 0.033 | 0.115 | 0.122 | 0.082 | -0.089 |
| H ( 45) | 0.04 | 0.12 | 0.128 | 0.08 | -0.088 |
| H ( 46) | 0.026 | 0.136 | 0.141 | 0.11 | -0.115 |
| H ( 47) | -0.011 | 0.191 | 0.189 | 0.202 | -0.2 |
| H ( 48) | 0.026 | 0.153 | 0.158 | 0.127 | -0.132 |
| H ( 49) | 0.026 | 0.162 | 0.167 | 0.136 | -0.141 |
| H ( 50) | 0.021 | 0.159 | 0.163 | 0.138 | -0.142 |
| Cl( 51) | 0.361 | -0.667 | -0.594 | -1.028 | 0.955 |

**Table S2** Fukui indices for the electrophilic and nucleophilic attacks on R_10_-IL

| atom | N0 | N+1 | N-1 | f+ | f- |
| --- | --- | --- | --- | --- | --- |
| C ( 1) | 0.079 | 0.097 | 0.113 | 0.018 | -0.034 |
| N ( 2) | 0.012 | -0.219 | -0.217 | -0.231 | 0.229 |
| C ( 3) | 0.027 | -0.028 | -0.022 | -0.055 | 0.049 |
| C ( 4) | 0.027 | -0.028 | -0.023 | -0.055 | 0.05 |
| N ( 5) | 0.015 | -0.223 | -0.22 | -0.238 | 0.235 |
| C ( 6) | -0.017 | -0.259 | -0.262 | -0.242 | 0.245 |
| C ( 7) | 0.011 | 0.15 | 0.152 | 0.139 | -0.141 |
| C ( 8) | 0.015 | -0.191 | -0.188 | -0.206 | 0.203 |
| C ( 9) | 0.012 | -0.185 | -0.183 | -0.197 | 0.195 |
| C ( 10) | 0.018 | 0.172 | 0.176 | 0.154 | -0.158 |
| C ( 11) | 0.017 | -0.187 | -0.183 | -0.204 | 0.2 |
| C ( 12) | 0.007 | -0.214 | -0.213 | -0.221 | 0.22 |
| C ( 13) | -0.01 | -0.512 | -0.514 | -0.502 | 0.504 |
| C ( 14) | -0.016 | -0.193 | -0.196 | -0.177 | 0.18 |
| C ( 15) | -0.009 | -0.275 | -0.277 | -0.266 | 0.268 |
| C ( 16) | -0.004 | -0.272 | -0.273 | -0.268 | 0.269 |
| C ( 17) | -0.005 | -0.265 | -0.266 | -0.26 | 0.261 |
| C ( 18) | -0.003 | -0.262 | -0.263 | -0.259 | 0.26 |
| C ( 19) | -0.003 | -0.264 | -0.264 | -0.261 | 0.261 |
| C ( 20) | -0.002 | -0.261 | -0.261 | -0.259 | 0.259 |
| C ( 21) | -0.002 | -0.26 | -0.26 | -0.258 | 0.258 |
| C ( 22) | -0.002 | -0.257 | -0.257 | -0.255 | 0.255 |
| C ( 23) | -0.001 | -0.43 | -0.431 | -0.429 | 0.43 |
| H ( 24) | 0.014 | 0.246 | 0.248 | 0.232 | -0.234 |
| H ( 25) | 0.044 | 0.155 | 0.164 | 0.111 | -0.12 |
| H ( 26) | 0.05 | 0.152 | 0.162 | 0.102 | -0.112 |
| H ( 27) | 0.043 | 0.164 | 0.173 | 0.121 | -0.13 |
| H ( 28) | 0.019 | 0.203 | 0.207 | 0.184 | -0.188 |
| H ( 29) | 0.034 | 0.116 | 0.123 | 0.082 | -0.089 |
| H ( 30) | 0.04 | 0.12 | 0.128 | 0.08 | -0.088 |
| H ( 31) | 0.028 | 0.134 | 0.14 | 0.106 | -0.112 |
| H ( 32) | -0.011 | 0.194 | 0.192 | 0.205 | -0.203 |
| H ( 33) | 0.025 | 0.149 | 0.154 | 0.124 | -0.129 |
| H ( 34) | 0.023 | 0.16 | 0.165 | 0.137 | -0.142 |
| H ( 35) | 0.026 | 0.163 | 0.168 | 0.137 | -0.142 |
| H ( 36) | 0.036 | 0.157 | 0.164 | 0.121 | -0.128 |
| H ( 37) | 0.007 | 0.219 | 0.221 | 0.212 | -0.214 |
| H ( 38) | 0.023 | 0.135 | 0.14 | 0.112 | -0.117 |
| H ( 39) | 0.002 | 0.159 | 0.159 | 0.157 | -0.157 |
| H ( 40) | -0.002 | 0.161 | 0.161 | 0.163 | -0.163 |
| H ( 41) | 0.015 | 0.134 | 0.137 | 0.119 | -0.122 |
| H ( 42) | 0.011 | 0.13 | 0.133 | 0.119 | -0.122 |
| H ( 43) | 0.004 | 0.137 | 0.138 | 0.133 | -0.134 |
| H ( 44) | 0.002 | 0.139 | 0.139 | 0.137 | -0.137 |
| H ( 45) | 0.008 | 0.132 | 0.133 | 0.124 | -0.125 |
| H ( 46) | 0.006 | 0.13 | 0.131 | 0.124 | -0.125 |
| H ( 47) | 0.002 | 0.134 | 0.134 | 0.132 | -0.132 |
| H ( 48) | 0.002 | 0.134 | 0.134 | 0.132 | -0.132 |
| H ( 49) | 0.004 | 0.131 | 0.132 | 0.127 | -0.128 |
| H ( 50) | 0.003 | 0.13 | 0.13 | 0.127 | -0.127 |
| H ( 51) | 0.002 | 0.13 | 0.131 | 0.128 | -0.129 |
| H ( 52) | 0.002 | 0.133 | 0.133 | 0.131 | -0.131 |
| H ( 53) | 0.003 | 0.131 | 0.132 | 0.128 | -0.129 |
| H ( 54) | 0.003 | 0.14 | 0.14 | 0.137 | -0.137 |
| H ( 55) | 0.002 | 0.141 | 0.141 | 0.139 | -0.139 |
| H ( 56) | 0.007 | 0.141 | 0.142 | 0.134 | -0.135 |
| Cl( 57) | 0.357 | -0.667 | -0.596 | -1.024 | 0.953 |

**Table S3** Fukui indices for the electrophilic and nucleophilic attacks on R_12_-IL

| Atom No. | N0 | N+1 | N-1 | f+ | f- |
| --- | --- | --- | --- | --- | --- |
| C ( 1) | 0.079 | 0.096 | 0.112 | 0.017 | -0.033 |
| N ( 2) | 0.011 | -0.225 | -0.223 | -0.236 | 0.234 |
| C ( 3) | 0.024 | -0.025 | -0.02 | -0.049 | 0.044 |
| C ( 4) | 0.031 | -0.033 | -0.027 | -0.064 | 0.058 |
| N ( 5) | 0.017 | -0.229 | -0.225 | -0.246 | 0.242 |
| C ( 6) | -0.014 | -0.255 | -0.258 | -0.241 | 0.244 |
| C ( 7) | 0.011 | 0.138 | 0.14 | 0.127 | -0.129 |
| C ( 8) | 0.015 | -0.191 | -0.188 | -0.206 | 0.203 |
| C ( 9) | 0.014 | -0.187 | -0.184 | -0.201 | 0.198 |
| C ( 10) | 0.018 | 0.171 | 0.175 | 0.153 | -0.157 |
| C ( 11) | 0.017 | -0.184 | -0.181 | -0.201 | 0.198 |
| C ( 12) | 0.005 | -0.2 | -0.199 | -0.205 | 0.204 |
| C ( 13) | -0.01 | -0.512 | -0.514 | -0.502 | 0.504 |
| C ( 14) | -0.018 | -0.18 | -0.183 | -0.162 | 0.165 |
| C ( 15) | -0.008 | -0.272 | -0.273 | -0.264 | 0.265 |
| C ( 16) | -0.005 | -0.276 | -0.277 | -0.271 | 0.272 |
| C ( 17) | -0.004 | -0.263 | -0.264 | -0.259 | 0.26 |
| C ( 18) | -0.003 | -0.265 | -0.265 | -0.262 | 0.262 |
| C ( 19) | -0.002 | -0.263 | -0.264 | -0.261 | 0.262 |
| C ( 20) | -0.002 | -0.262 | -0.263 | -0.26 | 0.261 |
| C ( 21) | -0.001 | -0.262 | -0.263 | -0.261 | 0.262 |
| C ( 22) | -0.001 | -0.261 | -0.261 | -0.26 | 0.26 |
| C ( 23) | -0.001 | -0.259 | -0.26 | -0.258 | 0.259 |
| C ( 24) | -0.001 | -0.257 | -0.257 | -0.256 | 0.256 |
| C ( 25) | -0.001 | -0.43 | -0.43 | -0.429 | 0.429 |
| H ( 26) | 0.013 | 0.249 | 0.252 | 0.236 | -0.239 |
| H ( 27) | 0.042 | 0.159 | 0.167 | 0.117 | -0.125 |
| H ( 28) | 0.05 | 0.151 | 0.161 | 0.101 | -0.111 |
| H ( 29) | 0.044 | 0.163 | 0.172 | 0.119 | -0.128 |
| H ( 30) | 0.013 | 0.215 | 0.217 | 0.202 | -0.204 |
| H ( 31) | 0.035 | 0.118 | 0.125 | 0.083 | -0.09 |
| H ( 32) | 0.041 | 0.12 | 0.128 | 0.079 | -0.087 |
| H ( 33) | 0.03 | 0.13 | 0.137 | 0.1 | -0.107 |
| H ( 34) | -0.007 | 0.186 | 0.184 | 0.193 | -0.191 |
| H ( 35) | 0.025 | 0.15 | 0.155 | 0.125 | -0.13 |
| H ( 36) | 0.022 | 0.157 | 0.162 | 0.135 | -0.14 |
| H ( 37) | 0.028 | 0.164 | 0.17 | 0.136 | -0.142 |
| H ( 38) | 0.033 | 0.163 | 0.17 | 0.13 | -0.137 |
| H ( 39) | 0.006 | 0.209 | 0.211 | 0.203 | -0.205 |
| H ( 40) | 0.019 | 0.138 | 0.142 | 0.119 | -0.123 |
| H ( 41) | 0.007 | 0.157 | 0.158 | 0.15 | -0.151 |
| H ( 42) | 0.003 | 0.155 | 0.156 | 0.152 | -0.153 |
| H ( 43) | 0.011 | 0.137 | 0.139 | 0.126 | -0.128 |
| H ( 44) | 0.009 | 0.132 | 0.134 | 0.123 | -0.125 |
| H ( 45) | 0.007 | 0.137 | 0.138 | 0.13 | -0.131 |
| H ( 46) | 0.003 | 0.139 | 0.139 | 0.136 | -0.136 |
| H ( 47) | 0.006 | 0.133 | 0.134 | 0.127 | -0.128 |
| H ( 48) | 0.005 | 0.131 | 0.132 | 0.126 | -0.127 |
| H ( 49) | 0.003 | 0.134 | 0.134 | 0.131 | -0.131 |
| H ( 50) | 0.001 | 0.134 | 0.134 | 0.133 | -0.133 |
| H ( 51) | 0.004 | 0.132 | 0.132 | 0.128 | -0.128 |
| H ( 52) | 0.003 | 0.13 | 0.131 | 0.127 | -0.128 |
| H ( 53) | 0.002 | 0.132 | 0.133 | 0.13 | -0.131 |
| H ( 54) | 0.001 | 0.132 | 0.132 | 0.131 | -0.131 |
| H ( 55) | 0.002 | 0.131 | 0.131 | 0.129 | -0.129 |
| H ( 56) | 0.002 | 0.13 | 0.13 | 0.128 | -0.128 |
| H ( 57) | 0.001 | 0.131 | 0.131 | 0.13 | -0.13 |
| H ( 58) | 0.001 | 0.132 | 0.132 | 0.131 | -0.131 |
| H ( 59) | 0.002 | 0.131 | 0.132 | 0.129 | -0.13 |
| H ( 60) | 0.002 | 0.14 | 0.14 | 0.138 | -0.138 |
| H ( 61) | 0.001 | 0.14 | 0.141 | 0.139 | -0.14 |
| H ( 62) | 0.005 | 0.141 | 0.142 | 0.136 | -0.137 |
| Cl( 63) | 0.359 | -0.677 | -0.606 | -1.036 | 0.965 |
